# HBimmCue: A Versatile Fluorescent Probe for Multi‐Scale Imaging of Lipid Polarity and Membrane Order in Inner Mitochondrial Membrane

**DOI:** 10.1002/advs.202414343

**Published:** 2025-02-09

**Authors:** Shu Gao, Jing Sun, Yiwei Hou, Xichuan Ge, Ming Shi, Hongxi Zheng, Yan Zhang, Meiqi Li, Baoxiang Gao, Peng Xi

**Affiliations:** ^1^ Department of Biomedical Engineering College of Future Technology Peking University Beijing 100871 P. R. China; ^2^ Key Laboratory of Analytical Science and Technology of Hebei Province College of Chemistry and Material Science Hebei University Baoding 071002 P. R. China; ^3^ School of Life Sciences Peking University Beijing 100871 P. R. China

**Keywords:** fluorescence lifetime imaging microscopy, HBimmCue, inner mitochondrial membrane, lipid polarity

## Abstract

Mitochondrial membrane environmental dynamics are crucial for understanding function, yet high‐resolution observation remains challenging. Here, HBimmCue is introduced as a fluorescent probe localized to inner mitochondrial membrane (IMM) that reports lipid polarity and membrane order changes, which correlate with cellular respiration levels. Using HBimmCue and fluorescence lifetime imaging microscopy (FLIM), IMM lipid heterogeneity is uncovered across scales, from nanoscale structures within individual mitochondria to mouse pre‐implantation embryos. At the sub‐organelle level, stimulated emission depletion (STED)‐FLIM imaging highlights nanoscale polarity variations within the IMM. At the sub‐cellular and cellular level, reduced IMM lipid polarity is observed in damaged mitochondria marked for lysosomal degradation and distinct IMM lipid distributions are identified in neurons and disease models. Additionally, metabolic dysfunction associated with oocytes aging and metabolic reprogramming from zygote to blastocyst is detected. Together, the work demonstrates the broad applicability of HBimmCue, offering a new paradigm for investigating lipid polarity and respiration level at multiple scales.

## Graphical abstract

HBimmCue is introduced as a fluorescent probe that detects lipid polarity changes in the inner mitochondrial membrane (IMM), reflecting cellular respiration levels. Using fluorescence lifetime imaging (FLIM), IMM lipid heterogeneity is uncovered across various scales, from nanoscale structures in mitochondria to cellular and developmental models, revealing insights into mitochondrial function, damage, and metabolic reprogramming.

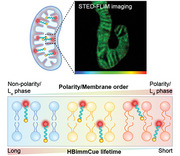

## 1 Introduction

Mitochondria are the powerhouses of cells, and mitochondria remodeling is essential for maintaining cellular metabolic plasticity, allowing cells to adapt to various changes.^[^ ^1^ ^]^ Structurally, mitochondria are enclosed by two lipid bilayer membranes: the outer mitochondrial membrane (OMM) and the inner mitochondrial membrane (IMM).^[^ ^1^, ^2^ ^]^ The OMM facilitates material exchange between the mitochondria and the cytosol, while the IMM is the site for the electron transport chain (ETC) and ATP synthesis. The IMM, in particular, exhibits remarkable complexity, with two main sub‐compartments: the inner boundary membrane (IBM) and cristae, which have distinct lipid compositions and functions.^[^ ^3^ ^]^ Phosphatidylcholine (PC), phosphatidylethanolamine (PE) and mitochondrial‐specific lipid cardiolipin (CL) are the major phospholipids in IMM.^[^ ^4^ ^]^ Specifically, PE and CL are presumably highly concentrated in cristae, which are predicted to directly influence membrane curvature, the structure of the cristae, and the efficient functioning of mitochondria.^[^ ^5^ ^]^

Understanding the IMM lipid heterogeneity and the roles of distinct lipids in crista organization and dynamics is of great interest. Separating mitochondria and performing mass spectrometry‐based lipidomic analysis is a common method for identifying lipid components in mitochondria.^[^ ^6^ ^]^ However, this approach has its limitations: on the one hand, it cannot achieve in situ identification of lipid components; on the other hand, the experimental protocols used for mitochondrial membrane separation, as well as the purity of the isolated fractions, can significantly affect the identification of lipid components.

Recent advances in fluorescence‐based technologies have significantly improved the ability to investigate lipid dynamics and heterogeneities across cell membranes.^[^ ^7^ ^]^ Techniques such as fluorescence correlation spectrum (FCS),^[^ ^8^ ^]^ fluorescence lifetime imaging microscopy (FLIM),^[^ ^9^ ^]^ fluorescence recovery after photobleaching (FRAP),^[^ ^10^ ^]^ single particle tracking,^[^ ^11^ ^]^ homo‐FRET^[^ ^12^ ^]^ and fluorescence polarization^[^ ^13^ ^]^ have been developed to probe various aspects of membrane dynamics with high sensitivity. Among these, FLIM imaging has emerged as a particularly powerful tool for investigating the heterogeneity of lipid membranes due to its sensitivity to environmental changes, independent of fluorophore concentration.^[^ ^14^ ^]^ Combining with environmentally sensitive fluorescent probes, FLIM can rapidly detect multiple properties of lipid membranes in situ, such as polarity, order, viscosity, etc.^[^ ^15^ ^]^

However, despite these advances, environmentally sensitive fluorescent probes remain scarce that specifically target IMM and can be applied across a wide range of biological samples, including cell lines, primary cells, and organisms (Table S1, Supporting Information).^[^ ^15^, ^16^ ^]^ Moreover, the resolution of FLIM imaging is limited by the optical diffraction limit, which hinders the ability to observe fluorescence lifetime distributions at the nanoscale, particularly within the IMM of living cells. While efforts have been made to enhance the resolution of FLIM by combining it with super‐resolution imaging techniques, such as single‐molecule imaging or image scanning microscopy,^[^ ^17^ ^]^ these approaches are still not sufficient for achieving nanoscale resolution across the entire IMM in real‐time living systems.

To address these challenges, in this work, we introduce HBimmCue, a novel near‐infrared, IMM‐targeting fluorescent probe. HBimmCue is sensitive to the lipid polarity and membrane order of IMM, which correlate with cellular respiration level. This probe enables ultra‐high resolution, cross‐scale observation of lipid heterogeneity, from sub‐organelle structures within individual mitochondria to whole mouse embryos. By leveraging HBimmCue, we have gained unprecedented insights into the spatial and functional diversity of mitochondrial membranes across a range of biological systems, laying the foundation for deeper exploration of mitochondrial dynamics in health and disease.

## 2 Results

### 2.1 Fluorescence Lifetime of HBimmCue Sensitively Responds to Polarity and Membrane Order

**Figure 1:**
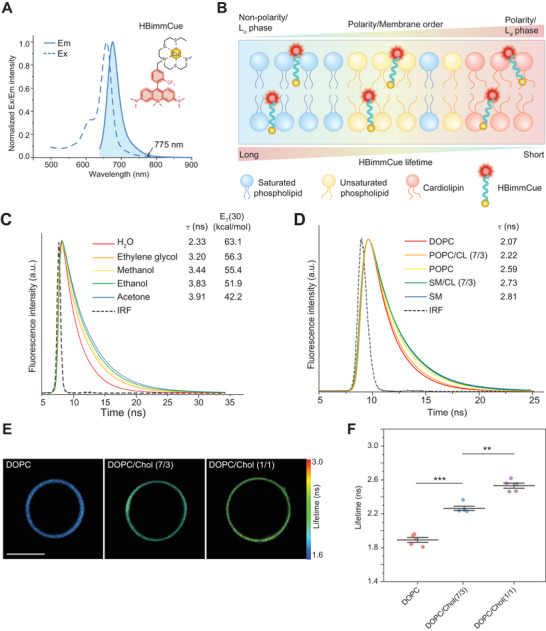
Polarity and membrane order response of HBimmCue fluorescence lifetimes. A,B) Schematics depicting the structure and functioning of HBimmCue as a sensitive reporter of polarity and membrane order. C) Time‐resolved fluorescence decays of HBimmCue in solvents of different polarity. The instrument response function (IRF) is presented as a dashed line (black). D) Time‐resolved fluorescence decays of HBimmCue in giant unilamellar vesicles (GUVs) solutions composed of 1,2‐dioleoyl‐sn‐glycero‐3‐phosphocholine (DOPC), 1‐Palmitoyl‐2‐oleoyl‐sn‐glycero‐3‐phosphorylcholine (POPC), sphingomyelin (SM) and cardiolipin (CL). The instrument response function (IRF) is presented as a dashed line (black). E) Representative FLIM images of GUVs composed of different molar ratios of DOPC and cholesterol (Chol). Scale bar, 5 µm. F) Fluorescence lifetimes of HBimmCue in indicated GUVs. N = 5 samples per group were used for statistical comparison. Two‐tailed *T*‐test for the statistic calculation. ^*^ *P* < 0.05 , ^**^ *P* < 0.01, ^***^ *P* < 0.001. Error bars represent the mean values ± standard deviation (S.D.) of indicated independent experiments.

To visualize the polarity and membrane order of IMM in cells, we introduced a near‐infrared fluorescence probe, **HBimmCue**, with an excitation wavelength maximum of 660 nm and an emission maximum of 678 nm (**Figure** **1**A). As a positively charged lipophilic molecule, HBimmCue targets the mitochondrial inner membrane through electrostatic interaction with the transmembrane potential. The long hydrophobic thioether chain of Bis(2‐((2‐(Ethylthio)ethyl)‐Thio)ethyl)Amine (BETA) group enhances lipophilicity, ensuring high specificity for the hydrophobic environment of the inner mitochondrial membrane.

In vitro studies demonstrated that the fluorescence lifetime of HBimmCue progressively increased in buffers with decreasing polarity, ranging from 2.33 ns in water (H₂O) to 3.91 ns in acetone, following a polarity gradient through ethylene glycol, methanol and ethanol (Figure 1C). We also tested other environmental factors that may affect the fluorescence lifetime of HBimmCue. Notably, in a water/glycerol mixture, the fluorescence lifetime of HBimmCue increased as the proportion of glycerol increased (Figure S1A, Supporting Information). However, no clear correlation was observed between the solvent composition and HBimmCue fluorescence lifetime in an ethylene glycol/glycerol mixture (Figure S1B, Supporting Information). Given the differences in polarity between water (E_T_(30) = 63.1 kcal mol^−1^, viscosity 1 mPa·s, 25 °C) and glycerol (E_T_(30) = 56.1 kcal mol^−1^, viscosity 1500 mPa·s, 25 °C), but comparable polarities between ethylene glycol (E_T_(30) = 56.3 kcal mol^−1^, viscosity 16.1 mPa·s, 25 °C) and glycerol, these results indicated that the fluorescence lifetime of HBimmCue primarily responds to solvent polarity rather than viscosity. Moreover, other environmental factors, such as pH and reactive oxygen species (ROS), had little or no effect on the fluorescence lifetime of HBimmCue (Figure S1C,D, Supporting Information). We reasoned that the polarity sensitivity of HBimmCue fluorescence lifetime is due to the following several factors. First, the polarity affects the photoinduced electron transfer (PET) process. In polar environments, HBimmCue has lower binding Cu(I) ability,^[^ ^18^ ^]^ leading to faster non‐radiative relaxation and a shorter lifetime. Additionally, the increased solvent polarity enhances solvent‐molecule interactions, promoting non‐radiative decay pathways such as solvent relaxation and internal conversion.^[^ ^19^ ^]^ As a result, the overall fluorescence lifetime decreases in more polar solvents.

The lower polarity inside the membrane is a result of the tighter packing of hydrophobic lipid tails, and indicates the liquid order phase (Lo), while the higher polarity indicates the liquid disordered phase (Ld).^[^ ^20^ ^]^ Thus, we further assessed the ability of HBimmCue to report changes in lipid environment in giant unilamellar vesicles (GUVs) composed of 1,2‐dioleoyl‐sn‐glycero‐3‐phosphocholine (DOPC), 1‐Palmitoyl‐2‐oleoyl‐sn‐glycero‐3‐phosphorylcholine (POPC)/Cardiolipin (7/3), POPC, sphingomyelin (SM)/Cardiolipin (7/3) and SM. The measured lifetimes were 2.07 ns, 2.22 ns, 2.59 ns, 2.73 ns, and 2.81 ns, respectively (Figure 1D). The results indicate that HBimmCue exhibits longer lifetimes in more ordered and less polar membrane environments (e.g., SM), while shorter lifetimes in disordered and more polar environments (e.g., DOPC). To further understand the probes’ sensitivity to membrane order and polarity, we adjusted the cholesterol (Chol) content in DOPC GUVs and measured the fluorescence lifetime. In DOPC, DOPC/Chol (7/3) and DOPC/Chol (5/5) GUVs, the fluorescence lifetimes increased progressively (Figure 1E,F). These findings collectively demonstrate that the fluorescence lifetime of HBimmCue is sensitive to lipids polarity and membrane order.

We also measured the excitation and emission spectrum of HBimmCue in different lipid microenvironments (Figure S2, Supporting Information). Our results indicate that the excitation spectra of GUVs containing different lipid compositions exhibited no significant differences. However, upon excitation at 520 nm, GUVs composed of POPC and 1,2‐dipalmitoyl‐sn‐glycero‐3‐phosphorylcholine (DPPC), characterized by tightly packed lipid environments, displayed slightly blue‐shifted emission bands, whereas GUVs composed of DOPC, with a more fluid lipid packing, exhibited slightly red‐shifted emission bands. These observations further enhanced the sensitivity of HBimmCue to lipid microenvironments.

Overall, these evidences revealed that HBimmCue showed a longer lifetime in low‐polarity, ordered‐membrane environments, and a shorter lifetime in high‐polarity, disordered‐membrane environments (Figure 1B).

### 2.2 HBimmCue Specifically Localizes to IMM and Reports Nanoscale Polarity Heterogeneity Across IMM

**Figure 2:**
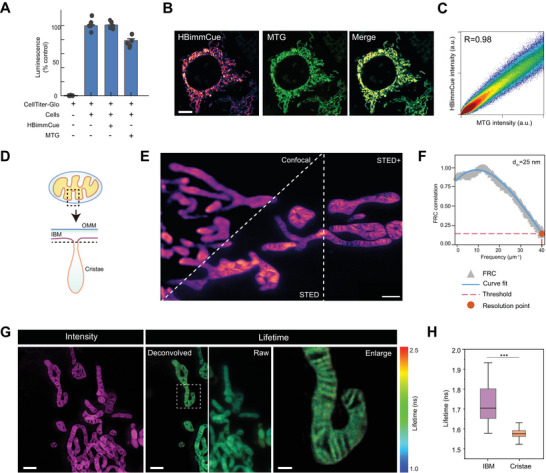
HBimmCue specifically localizes to IMM and reports nanoscale polarity heterogeneity across IMM. A) COS‐7 cells were labeled with 500 nM HBimmCue or MitoTrackerGreen (MTG) for 15 min and cell viability was measured with standard CellTiter‐Glo protocol (Methods). B) Representative confocal images of COS‐7 cells labeled with HBimmCue or MTG. Scale bar, 10 µm. C) Scatter plot of HBimmCue and MTG images in (B). Fourier correlation coefficient (R) was calculated with Coloc2 plugin in Fiji. D) Cartoon schematic of mitochondrial membranes. OMM, outer mitochondrial membrane; IBM, inner boundary membrane. E) Representative images of confocal, STED and STED+ (deconvolved with Huygens) results of living COS‐7 cells labeled with HBimmCue. Scale bar, 1 µm. F) Absolute resolution of STED obtained by Fourier Ring Correlation (FRC). G) Representative STED (left), STED‐FLIM (middle) and enlarged STED‐FLIM (right) results of COS‐7 cells labeled with HBimmCue. The raw STED‐FLIM image was generated directly by the LAX software of Leica. The deconvolved STED‐FLIM image was generated by superimposing fluorescence lifetime map to the MRA‐deconvolved intensity image. Scale bars, 1 µm in the left and middle panel, 200 nm in the right panel. H) Quantitative comparison of fluorescence lifetimes of HBimmCue in the IBM and cristae. n = 10 mitochondria were used for statistics. Two‐tailed *T*‐test for the statistic calculation. ^*^ *P* < 0.05, ^**^ *P* < 0.01, ^***^ *P* < 0.001. Error bars represent the mean values ± standard deviation (S.D.) of indicated independent experiments.

Next, we tested the cellular labeling performance of HBimmCue. Assessment of cellular viability using an ATP detection assay (CellTiter‐Glo 2.0) indicated the excellent biocompatibility of HBimmCue compared with the commonly used MitoTrackerGreen (MTG) fluorescence probes for mitochondria labeling (**Figure** **2**A). For cellular labeling, we labeled COS‐7 cells with 500 nmHBimmCue at 37 °C for 15 min, and replaced with fresh culture medium for imaging. HBimmCue showed highly correlated co‐localization with MTG and COX8A, an inner mitochondrial protein marker (Figure 2B,C; Figure S3, Supporting Information).

We also tested the co‐localization of HBimmCue with probes targeted other organelles (Lysosome, LysoTrackerGreen; Endoplasmic reticulum (ER), ER‐Tracker Green) (Figure S4A, Supporting Information), as well as in other cell types, including U‐2OS and BSC1cell lines (Figure S4B, Supporting Information), reaffirming the specificity of HBimmCue for mitochondrial labeling in various cell lines. Long‐term time‐lapse confocal imaging experiments revealed the superior photostability of HBimmCue compared to commercial dyes excited at different wavelengths, including MTG (488 nm), Tetramethylrhodamine methyl ester perchlorate (TMRM, 561 nm), and MitoTracker Deep Red (MTDR, 647 nm) (Figure S4C–F; Movie S1, Supporting Information). After 1000 s of imaging, HBimmCue retained ≈90% of its initial brightness and maintained excellent mitochondrial specificity. In contrast, MTDR showed a significant brightness decrease to 60% of its initial value, with noticeable signal diffusion to the endoplasmic reticulum.

Due to its strong lipophilicity, HBimmCue exhibited low sensitivity to mitochondrial membrane potential. In FCCP‐treated COS‐7 cells, TMRM fluorescence nearly disappeared, while HBimmCue retained almost unchanged brightness (Figure S5 and Movie S2, Supporting Information). In 4% PFA‐fixed COS‐7 cells, HBimmCue partially remained on the mitochondrial inner membrane, preserving some fluorescence signal. However, a fraction of the probe is redistributed to the endoplasmic reticulum and other membranous organelles (Figure S6, Supporting Information). Thus, although HBimmCue is capable of imaging after cell fixation, considering the labeling specificity and fluorescence intensity, we do not recommend the utilization of HBimmCue after fixation, which is analogous to other commercial mitochondrial probes.

To figure out the effect of concentration on the fluorescence lifetime of HBimmCue, we measured the fluorescence decay curves of HBimmCue in aqueous solution and GUVs composed of DOPC at different concentrations (Figure S7, Supporting Information). The results showed that HBimmCue exhibited nearly the same fluorescence lifetime across these different samples, indicating that the fluorescence lifetime of HBimmCue is not sensitive to the concentration within this range. Additionally, we labeled COS‐7 cells and U‐2OS cells with 200 and 500 nM HBimmCue at 37 °C for 15 min, followed by fluorescence lifetime imaging (Figure S8A,C, Supporting Information). We observed no significant differences in the average fluorescence lifetimes between cells labeled with 200 and 500 nM HBimmCue (Figure S8B,D, Supporting Information). These results consistently demonstrate that the fluorescence lifetime of HBimmCue is not sensitive to concentration changes under the tested conditions. Moreover, all the identical samples used for inter‐group comparison in our study were labeled using the same protocol to avoid any labeling artifacts to the results.

To further assess the sub‐organelle localization of HBimmCue (Figure 2D), COS‐7 cells labeled with HBimmCue were used for STED imaging, with subsequent processing with the Huygens deconvolution algorithm, termed STED+ (Figure 2E).^[^ ^21^ ^]^ The ultra‐structure of mitochondrial cristae was clearly visible, further indicating the high IMM specificity of HBimmCue. The absolute resolution of STED images, calculated by Fourier Ring Correlation (FRC) reached up to 25 nm^[^ ^22^ ^]^ (Figure 2F). Moreover, time‐lapse structured illumination microscopy (SIM) imaging further enabled us to visualize dynamic mitochondrial processes such as tubulation and fission, with clear cristae visible under SIM imaging conditions (Figure S9 and Movie S3, Supporting Information).

After confirming the ability of HBimmCue for super‐resolution imaging, we subsequently performed STED‐FLIM imaging to observe the different lipid compositions across IMM using the Leica Stellaris 8 system (Figure 2G). To enhance the resolution of the STED‐FLIM results without affecting the fluorescence lifetime values, we deconvolved the STED grayscale images and subsequently re‐mapped the fluorescence lifetime values onto the deconvolved grayscale images. This approach significantly improved the resolution of the STED‐FLIM images, enabling clearer observation of spatial differences in fluorescence lifetime distribution across IMM (Figure 2G). Interestingly, HBimmCue showed a longer lifetime in IBM than in cristae (Figure 2H), which may be attributed to the higher enrichment of cardiolipin in the cristae, leading to increased membrane polarity and disorder.^[^ ^4a^ ^]^ A recent study has also observed the higher IMM polarity in cristae by FLIM imaging, although they examined the lifetime distribution of the swollen mitochondrial at diffraction‐limited spatial resolution.^[^ ^15a^ ^]^

The above results demonstrated the excellent performance of HBimmCue in cellular IMM labeling and its compatibility with various super‐resolution imaging techniques. Combined with FLIM imaging and image post‐processing algorithms, it provides a powerful tool for studying the super‐resolved IMM lipid heterogeneity in distinct biological processes.

### 2.3 IMM Lipid Polarity Changes Charactered by FLIM Imaging of HBimmCue

**Figure 3:**
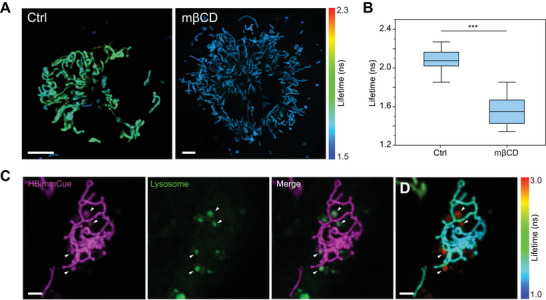
IMM lipid polarity and order changes charactered by FLIM imaging of HBimmCue. A) Representative FLIM images of control and 1 mM mβCD treated COS‐7 cells. Scale bar, 10 µm. B) Quantitative comparison of fluorescence lifetimes of control and 1 mM mβCD treated COS‐7 cells. n = 20 cells per group were used for statistics. C) COS‐7 cells co‐labeled with HBimmCue (magenta, IMM) and LysoView 488 (green, lysosomes). White triangles indicated HBimmCue+/LysoView+ double positive vesicles that indicated mitochondria removed by lysosomes. Scale bar, 10 µm. D) FLIM imaging of COS‐7 cells labeled with HBimmCue in (C). White triangles indicated HBimmCue+/LysoView+ double positive vesicles that showed significantly higher fluorescence lifetimes. Two‐tailed *T*‐test for the statistic calculation. ^*^ *P* < 0.05, ^**^ *P* < 0.01, ^***^ *P* < 0.001. Error bars represent the mean values ± standard deviation (S.D.) of indicated independent experiments.

We subsequently validated the ability of HBimmCue to visualize the changes in IMM lipid polarity through the modulation of cholesterol contents using the cholesterol‐sequestering agent methyl‐β‐cyclodextrin (mβCD), which reducing the cholesterol content and thereby increasing membrane polarity and reducing membrane order. After incubating COS‐7 cells in 1 mm mβCD for 3 h, we subjected mβCD‐treated and controlled COS‐7 cells for HBimmCue labeling and FLIM imaging, respectively. We observed a relatively shorter lifetime in mβCD‐treated cells compared to the control (**Figure** **3**A,B), which indicated the increase in IMM lipid polarity as well as the decrease in IMM membrane order in mβCD‐treated cells.

We further explored the sensitivity of HBimmCue for reporting intracellular mitochondrial lipid heterogeneity. Mitochondria homeostasis is maintained by several quality control pathways, such as the removal of entire damaged mitochondria through mitophagy, as well as mitochondria remodeling by mitochondria‐lysosome‐related organelles.^[^ ^23^ ^]^ A recent study has also revealed that lysosomes drive the piecemeal removal of the mitochondrial inner membrane.^[^ ^24^ ^]^ The removal of unhealthy mitochondria was found to be essential for maintaining cellular fitness. Thus, we tended to explore the changes in IMM lipid properties in damaged mitochondria removed by lysosomes. We co‐labeled COS‐7 cells with HBimmCue and LysoView488 for IMM and lysosome labeling, respectively. We observed HBimmCue^+^/LysoView^+^ double positive vesicles that indicated mitochondria removed by lysosomes (Figure 3C). Surprisingly, in the FLIM imaging results of HBimmCue, the probe showed a significantly longer lifetime in the HBimmCue^+^/LysoView^+^ double positive vesicles than in other mitochondria (Figure 3D). This indicated the decrease of IMM lipid polarity in the lysosomal‐cleared mitochondria, which may be due to the peroxidation of the unsaturated fatty acids caused by the excess production of ROS in the damaged mitochondria.^[^ ^15a^ ^]^

### 2.4 Differential Distribution of IMM Lipid Polarity in Neurons and Mitochondrial Diseases

To further assess the versatility of HBimmCue, we applied it to different types of biological samples. Mitochondria play a crucial role in the neuron system, and abnormalities in mitochondria are associated with various neurological disorders, such as neurodegenerative diseases, chronic progressive external ophthalmolegia (CPEO) and mitochondrial encephalopathy, lactic acidosis and stroke‐like episodes (MELAS) diseases.^[^ ^23^, ^25^ ^]^ Thus, we aimed to investigate the mitochondrial membrane properties in neurons and mitochondrial diseases.

**Figure 4:**
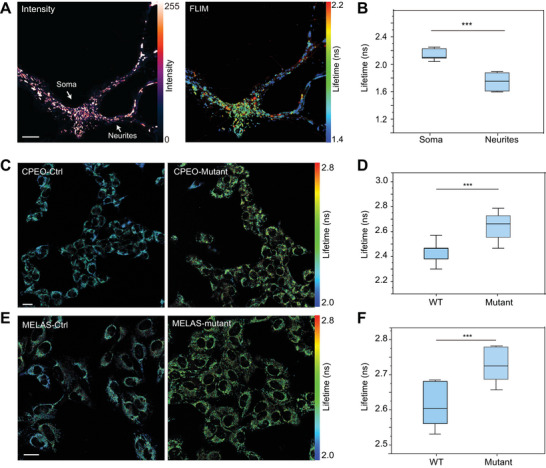
FLIM imaging reveals polarity heterogeneity in neuron and mitochondrial‐disease associated cells. A) Representative images of confocal (left) and FLIM (right) imaging results of mouse hippocampus neurons labeled with HBimmCue. Scale bars: 10 µm. B) Quantitative comparison of mean fluorescence lifetime of HBimmCue in the soma and neurites. n = 10 cells in each group were used for statistics. C) Representative images of FLIM imaging results of WT chronic progressive external ophthalmoplegia (CPEO) cells (left) and mtDNA mutant CPEO cells (right) labeled with HBimmCue. Scale bars: 50 µm. D) Quantitative comparison of mean fluorescence lifetime of HBimmCue in WT and mutant CPEO cells. n = 10 cells in each group were used for statistics. E) Representative images of confocal‐FLIM imaging results of WT cells (left) and 43B mtDNA mutant MELAS cells (right) labeled with HBimmCue. Scale bars: 50 µm. F) Quantitative comparison of mean fluorescence lifetime of HBimmCue in WT and mutant MELAS cells. n = 10 cells in each group were used for statistics. Two‐tailed *T*‐test for the statistic calculation. ^*^ *P* < 0.05, ^**^ *P* < 0.01, ^***^ *P* < 0.001. Error bars represent the mean values ± standard deviation (S.D.) of indicated independent experiments.

We co‐labeled mouse hippocampus neurons at day in vitro 7 (DIV7) with HBimmCue and MTG, followed by confocal and FLIM imaging. The results showed that HBimmCue specifically localized to mitochondria in neurons. Notably, we observed a significantly longer fluorescence lifetime of HBimmCue in somata compared to neurites (**Figure** **4**A; Figure S10A,B, Supporting Information), which demonstrated the differential IMM lipid polarity and membrane order between neuron somata and neurites. However, no significant differences were observed neither in the fluorescence intensity of HBimmCue and MTG, nor in the fluorescence lifetime of MTG, between somata and neurites (Figure S10B–D, Supporting Information). Recent research has indicated that neuronal somata exhibit higher levels of aerobic glycolysis and lower levels of OXPHOS than terminals,^[^ ^26^ ^]^ and we assumed that the different metabolism states in somata and neurites may contribute to the variation in IMM lipid composition and HBimmCue fluorescence lifetime.

It has been revealed that mitochondrial membrane lipids influence oxidative phosphorylation (OXPHOS) levels through complex mechanisms,^[^ ^27^ ^]^ and thus we tended to test the correlation between cellular respiration level and fluorescence lifetime of HBimmCue. Auto‐fluorescence lifetime imaging of NAD(P)H is a marker‐free and robust approach to quantify the relative OXPHOS versus glycolysis levels, where higher NAD(P)H fluorescence lifetime indicates increased OXPHOS level.^[^ ^28^ ^]^ COS‐7 cells treated with Oligomycin A for the inhibition of mitochondrial respiration were subjected to two‐photon FLIM imaging (2p‐FLIM) of NAD(P)H and single‐photon FLIM imaging of HBimmCue. Notably, Oligomycin A treatment led to a clear decrease in cellular NAD(P)H lifetimes, while a significant increase in cellular HBimmCue lifetimes (Figure S11, Supporting Information), and OligomycinA itself did not have an effect on the probe's lifetime (Figure S12, Supporting Information). These results validated that the fluorescence lifetime of HBimmCue is a sensitive indicator of cell respiration level, whereas a higher cellular respiration level correlates with a shorter fluorescence lifetime of HBimmCue.

To further explore this, we used HBimmCue to characterize IMM properties in two mitochondrial diseases derived cell lines, including chronic progressive external ophthalmoplegia (CPEO) and mitochondrial encephalomyopathy (MELAS). CPEO is caused by mitochondria DNA deletion, while MELAS is caused by A3243G (43B) point‐mutation of mitochondria DNA.^[^ ^25^, ^29^ ^]^ The mtDNA mutations in these two disease models lead to defects in mitochondrial proteins, resulting in abnormalities in mitochondrial states and function. We labeled wild type (WT) CPEO cells and mutant CPEO cells with HBimmCue probes and performed FLIM imaging. HBimmCue showed a significantly longer fluorescence lifetime in mutant CPEO cells than in WT CPEO cells, indicating reduced lipid polarity in IMM (Figure 4C,D). Similarly, HBimmCue also showed a longer fluorescence lifetime in mutant MELAS cells compared with WT MELAS cells, suggesting lower IMM lipid polarity in mutant MELAS as well (Figure 4E,F). These observations suggested a significant decrease in IMM lipid polarity and OXPHOS levels in these two mitochondrial diseases. We hypothesize that the decreased polarity and increase of the lipid order in IMM in these mitochondrial diseases may hinder the diffusion and transport of respiration‐related metabolites across the membrane, potentially impairing mitochondrial function and reducing cellular respiration efficiency. This alteration could contribute to the pathophysiology of these disorders by affecting the cells’ ability to meet energy demands under stress, further disrupting neuronal function and cellular homeostasis.

### 2.5 Disruption of Mitochondrial Function in Aged Oocytes

Oocytes, the largest cell in multicellular organisms, harbor a high abundance of mitochondria, which are essential for their maturation and fertilization.^[^ ^30^ ^]^ Abnormal mitochondrial function is closely associated with ovarian decline and diminished oocyte quality in aging female mammals.^[^ ^31^ ^]^

**Figure 5:**
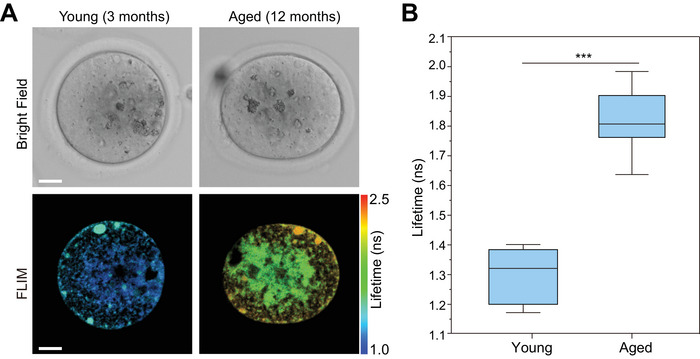
Disruption of mitochondrial function in aged oocytes. A) Representative images of bright‐field (upper) and FLIM (bottom) imaging results of mouse oocytes obtained from female mice at 3 and 12 months, respectively. Scale bars, 20 µm. B) Quantitative comparison of mean fluorescence lifetimes of HBimmCue in oocytes obtained from female mice at 3 and 12 months, respectively. n = 8 samples in each group were used for quantitative comparison. Two‐tailed *T*‐test for the statistic calculation. ^*^ *P* < 0.05, ^**^ *P* < 0.01, ^***^ *P* < 0.001. Error bars represent the mean values ± standard deviation (S.D.) of indicated independent experiments.

To determine whether FLIM imaging of HBimmCue can detect metabolic dysfunction during oocytes aging, we collected oocytes from female mice at 3 and 12 months of age and performed FLIM imaging of oocytes labeled with HBimmCue (**Figure** **5**A). In mouse oocytes, immature mitochondria appear spherical with few arched cristae.^[^ ^30^ ^]^ To evaluate the specificity of HBimmCue for mitochondrial labeling in oocytes, mitochondria were stained with MTG, and lipid droplets were labeled with Nile Red. HBimmCue showed high colocalization with MTG (Pearson's correlation coefficient *r* = 0.90) while nearly no colocalization with Nile Red (*r* = 0.23), demonstrating its specificity for mitochondrial labeling in oocytes (Figure S13, Supporting Information). The bright field images revealed no obvious morphological differences among oocytes from different age groups. In contrast, the fluorescence lifetime of HBimmCue was significantly different between young and old oocytes: the mean fluorescence lifetime was 1.29 ns in oocytes from 3‐month‐old mice, while 1.82 ns from 12‐month‐old mice, respectively (n = 8 in each group) (Figure 5B). The increase in fluorescence lifetime indicates the disrupted mitochondrial function in aged oocytes, consistent with the previously reported decline in NADH lifetime in aged oocytes.^[^ ^32^ ^]^

These findings collectively highlight the pronounced changes in mitochondria properties associated with aging in oocytes. Understanding these alterations is critical, as they may contribute to the overall decline in oocyte quality and fertility observed in older females. Our results underscore the importance of maintaining mitochondrial health for optimal reproductive function and provide a useful tool for the quality control of oocytes in the field of assisted reproduction technology.

### 2.6 Noninvasive Metabolic Profiling of Mouse Pre‐Implantation Embryos via FLIM Imaging of HBimmCue

**Figure 6:**
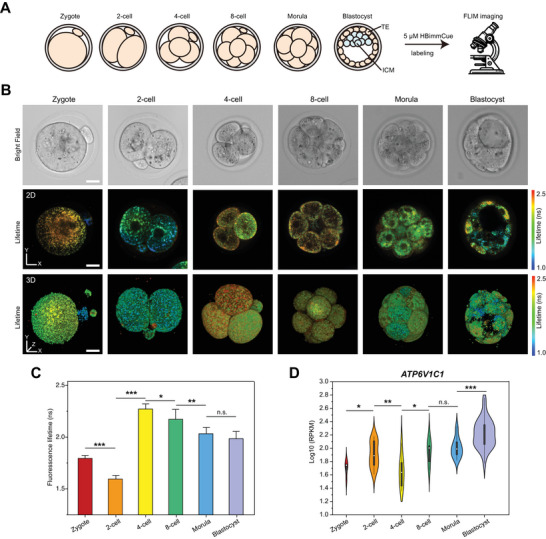
IMM lipid order changes during mouse pre‐implantation embryo development. A) Schematic of FLIM imaging experiments of mouse pre‐implantation embryos labeled by HBimmCue. B) Representative images of bright‐field (first row), 2D‐FLIM (second row) and 3D‐FLIM (third row) imaging results of mouse pre‐implantation embryos labeled with HBimmCue. Scale bars, 20 µm. C) Quantitative comparison of mean fluorescence lifetime of HBimmCue in mouse pre‐implantation embryos across different developmental stages. Zygotes (n = 8), 2‐cell (n = 10), 4‐cell (n = 6), 8‐cell (n = 5), morula (n = 8), and blastocyst (n = 7) embryos were used for quantitative comparison. D) Violin plots showing the expression level of *ATP6V1C1* in mouse pre‐implantation embryos. Zygotes (n = 4), 2‐cell embryo (n = 13), 4‐cell embryo (n = 7), 8‐cell embryos (n = 6), morula (n = 16), blastocyst (n = 31) cells were used for quantitative comparison. Two‐tailed *T*‐test for the statistic calculation. ^*^ *P* < 0.05, ^**^ *P* < 0.01, ^***^ *P* < 0.001. Error bars represent the mean values ± standard deviation (S.D.) of indicated independent experiments.

Metabolic reprogramming is essential for mammalian early embryo development,^[^ ^33^ ^]^ which is closely correlated with the changes in mitochondria properties. Thus, we applied HBimmCue probes for mouse pre‐implantation embryo labeling at zygote, 2‐cell, 4‐cell, 8‐cell, morula and blastocyst stages and then performed FLIM imaging (**Figure** **6**A). HBimmCue showed excellent permeability for mouse pre‐implantation embryo labeling, without any other sample processing methods, which guaranteed the viability of the embryos to the greatest extent. The imaging results of the pre‐implantation embryos revealed significant changes in the fluorescence lifetime of HBimmCue at different developmental stages (Figure 6B; Figure S14A and Movie S4, Supporting Information). A substantial decrease (*P* < 0.001) in fluorescence lifetime was observed between the zygote (n = 8) and 2‐cell stage (n = 10), followed by a sudden increase at the 4‐cell stage (n = 6, *P* < 0.001). A gradual decrease in fluorescence lifetime was seen between the 4‐cell stage and blastocyst stage (n = 8, *P* < 0.005) (Figure 6C). We proposed that the sudden decrease in fluorescence lifetime at the 2‐cell stage may reflect special characteristics of IMM during the zygotic genome activation (ZGA) process.^[^ ^33^ ^]^ The trend toward a decrease in fluorescence lifetime from the 4‐cell stage to the blastocyst stage may be necessary to support the changing metabolic demands of the pre‐implantation embryos. To test this hypothesis, we analyzed a public single‐cell RNA‐seq dataset obtained from mouse pre‐implantation embryos,^[^ ^34^ ^]^ and we found that the transcription level of *ATP6V1C1, ATP6V0B, and ATP5B*, three marker genes of OXPHOS, were increased from 4‐cell to blastocyst stage, which showed an inverse variation pattern compared with HBimmCue fluorescence lifetime (Figure 6D; Figure S14B,C, Supporting Information). This indicated that the fluorescence lifetime distribution patterns of HBimmCue were correlated with the metabolic states of mouse pre‐implantation embryos.

The first cell fate decision in early embryos is a crucial topic in developmental biology, which generates the inner cell mass (ICM) and trophoblast (TE) lineages. Recent single‐cell multi‐omics sequencing‐based or imaging‐based studies have shown that as early as the two‐cell stage, the two cells of the same embryo already exhibit different developmental potentials.^[^ ^35^ ^]^ Interestingly, we also observed differential cellular fluorescence lifetime distribution within the same embryo at 2‐cell and 4‐cell stages (Figure 6B). The differential fluorescence lifetime distribution in the same embryo may reflect the cellular heterogeneity and different developmental potential in the same embryo as early as a 2‐cell stage.^[^ ^35^, ^36^ ^]^ At the blastocyst stage, a striking difference in fluorescence lifetime was observed between the trophoblast (TE) cells and the inner cell mass (ICM), as well as between cells with the same identity. This suggested differential mitochondrial states between these cells, which may relate to their different metabolism states and developmental potential.

Together, the excellent tissue permeability of HBimmCue, along with its sensitivity to IMM lipid polarity and order, makes it a powerful, non‐invasive imaging tool for mitochondria profiling of entire tissues in physiological states. These findings provide valuable insights into how mitochondrial dynamics are intricately linked to metabolic demands and pre‐implantation developmental progress.

## 3 Conclusion

In summary, we utilized FLIM imaging to investigate the heterogeneity of IMM polarity and membrane order across various biological samples and at different scales. HBimmCue probe demonstrated a sensitive response to lipid polarity and membrane order, linked to the cellular respiration level. HBimmCue also exhibits excellent biocompatibility, tissue permeability, fluorescence brightness, and photostability, which make it compatible with advanced super‐resolution imaging techniques such as STED and SIM. These properties make HBimmCue a powerful tool for mitochondrial structural and functional imaging. At the nanoscale level, using STED‐FLIM imaging, we revealed distinct lipid polarity in different regions of IMM, and we supposed that this may reflect the distinct protein and lipid contents in IMM subdomains. The lower fluorescence lifetime in the cristae than in IBM may correlate with the active respiration processes across the cristae. Indeed, the dynamic sub‐compartmentalization of IMM has been previously reported using quantitative immunoelectron microscopy.^[^ ^37^ ^]^ Further advances in super‐resolution imaging technologies and environmental‐sensitive fluorescence probes would yield deeper insights into the relationship between the mitochondrial membrane environment and its structure and function.

At the subcellular and cellular level, we uncovered differences in IMM environments between damaged mitochondria cleared by lysosomes and those in a normal state. In mouse primary hippocampal neurons, we observed significant differences in mitochondrial membrane properties between the somata and neurites, which may correlate with different cellular respiration levels in these two regions. In two mitochondrial disease models, MELAS and CPEO, mutant cells exhibited increased IMM order as well as a decline in cellular respiration level. Moreover, we also observed the disrupted mitochondria function in aged mouse oocytes. This application highlights the potential of FLIM for use in oocyte quality control and reproductive assistance. Collectively, these findings clearly demonstrate the close relationship between IMM lipid polarity and mitochondrial function: the decline in mitochondrial membrane polarity and the increase in membrane order may disrupt the diffusion and transport of metabolites associated with the ETC, leading to reduced OXPHOS level.

At the organism level, we explored the remodeling of mitochondrial lipid polarity during mouse pre‐implantation embryo development. This remodeling enables the embryo to adapt to the changing metabolic and developmental demands at different stages. Compared to other methods, such as single‐cell multi‐omics sequencing,^[^ ^34^, ^38^ ^]^ or long‐term live imaging and lineage tracing,^[^ ^35^, ^39^ ^]^ FLIM imaging provides a simpler, widely applicable, and cost‐effective approach. Previous studies using the autofluorescence lifetimes of NAD(P)H and FAD have explored metabolic remodeling during early embryonic development, but these markers lack organelle specificity.^[^ ^40^ ^]^ Our work demonstrates the promising applications of FLIM combined with environmentally sensitive fluorescent probes in developmental biology research.

## 4 Experimental Section

### Ultraviolet Absorption and Fluorescence Emission Spectrometry Measurement

Stock solutions of HBimmCue were prepared in DMSO solvent at 5 mm. The absorption spectrum was measured using a Specord 210 plus spectrophotometer (Germany) in a 1 cm square quartz cuvette. The fluorescence emission spectrum was measured using FLS1000 Photoluminescence Spectrometer (England) in a 1 cm square quartz cuvette. All the measurements were performed in ultrapure water with the presence of saturated Cu(I) at room temperature. Cu(I) was delivered in the form of [Cu(MeCN)_4_[PF_6_]] from a 5 mM stock solution.

### Fluorescence Lifetime Measurement in Solution

Time‐resolved fluorescence spectra of HBimmCue in various buffers were measured using the FLS1000 Photoluminescence Spectrometer (England). Unless otherwise mentioned, all the decay profiles were fitted reasonably well with a single exponential function.

### Preparation and Lifetime Measurement of Giant Unilamellar Vesicles (GUVs)

1,2‐Dioleoyl‐sn‐glycero‐3‐phosphocholine (DOPC, Avanti), 1‐Palmitoyl‐2‐oleoyl‐sn‐glycero‐3‐phosphorylcholine (POPC, Avanti), 1,2‐Dipalmitoyl‐sn‐glycero‐3‐phosphorylcholine (DPPC, Avanti) and cardiolipin (CL, Avanti) were used for GUVs preparation. GUVs were prepared according to the protocol of Nanion Vesicle Prep Pro setup (Germany). Briefly, stock solutions (10 mM) of lipids in CHCl_3_ were warmed up for a few minutes at room temperature, then 20 µL lipid solution with 1% mol HBimmCue was put on the conducting side of the ITO coated slide and let the solvent evaporate for a few minutes. Grease an o‐ring and place it around the dry lipid film. Add 250 µL 1 M  sorbitol. Place the second ITO slide with the conductive side down on the top of the ring. Close the Vesicle Prep Chamber and connect to the VesiclePrep Station. Run the protocol (at 1 V with a 10 Hz frequency for 2 h at 25 °C). After 2 h the GUVs were formed. GUVs can be dissolved with 2 mL PBS for spectrum and fluorescence decay curve measurement using FLS1000 Photoluminescence Spectrometer (England), or directly used for FLIM imaging using the Leica Stellaris 8 microscopy.

### Cell Culture—*Cell lines*

COS‐7 cells (ATCC, USA), U‐2OS cells (ATCC, USA) and BSC1 cells (ATCC, USA) were cultured in Dulbecco's Modified Eagle's medium (Gibco, 11965‐092) supplemented with 10% (v/v) heat‐inactivated Fetal Bovine Serum (Thermofisher Scientific) and 1% (v/v) Penicillin‐streptomycin (ThermoFisher Scientific, 15140122). Cells were incubated in a sterile and humid incubator with 5% CO_2_ at 37 °C.

### Cell Culture—Primary Cells

Mouse hippocampus neurons were isolated from C57BL/6J mice within 12 h of birth and cell culture plate were pre‐coated with poly‐D‐Lysine (Gibco, A3890401), and were cultured in Neurobasal medium (Invitrogen, 21103049) supplemented with 1× B27 (Thermofisher Scientific, A3582801), 1× GlutaMax (Thermofisher Scientific, 35050061) and 1× Penicillin‐streptomycin (ThermoFisher Scientific, 15140122). Cell medium of hippocampus neurons was half‐exchanged every two days, and imaging experiments were performed at 7 days in vitro (DIV7).

### Cell Culture—Mitochondrial Diseases Derived Cell Lines

WT CPEO, mutant CPEO, WT MELAS and mutant MELAS cell lines were kind gifts from Prof. Yan Zhang. These four cell lines were cultured in Dulbecco's Modified Eagle's medium (Gibco, 11965‐092) supplemented with 50 µg/mL Uridine (HARVEYBIO, NL1121), 10% (v/v) heat‐inactivated Fetal Bovine Serum (Thermofisher Scientific) and 1% (v/v) Penicillin‐streptomycin (ThermoFisher Scientific, 15140122). All cell lines were authenticated to be free of mycoplasma contamination by PCR before usage in this study.

### Animals

All animal experiments were performed according to the protocols approved by the Institutional Animal Care and Use Committee of Peking University. All mice were maintained in pathogen‐free conditions in the Laboratory Animal Center of Peking University on a 12‐12 h light‐dark cycle and bred with a normal diet.

### Collection of Mouse Pre‐Implantation Embryos

For mouse oocyte collections, 3 and 12 months C57BL/6J female mice were superovulated by intraperitoneal injection of 7.5 IU of pregnant mare's serum gonadotropin (PMSG) (San‐Sheng pharmaceutical Co. Ltd) followed by 7.5 IU of human chorionic gonadotropin (HCG) (San‐Sheng pharmaceutical Co. Ltd) 40 h later. Oocytes were collected 14 hrs later after HCG injection. For mouse pre‐implantation embryo collections, 4‐weeks C57BL/6J female mice were superovulated by intraperitoneal injection of 7.5 IU of PMSG followed by 7.5 IU of HCG 40 h later, and mated with 2‐month‐old C57BL/6J male mice. Embryos at different developmental stages were collected at defined time periods after HCG administration: 14–16 h (Oocytes), 22–24 h (Zygotes), 40–48 h (2‐cell), 54‐46 h (4‐cell), 68–70 h (8‐cell), 78–80 h (morula) and 88–90 h (blastocytes). For oocytes and zygotes collection, a cumulus mass was flushed from the oviduct in M2 medium (Nanjing Aibei Biotechnology Co., Ltd) and transferred to Hyaluronidase solution (Nanjing Aibei Biotechnology Co., Ltd) and incubated at 37 °C for several minutes. The oocytes or zygotes were transferred to KSOM medium (Nanjing Aibei Biotechnology Co., Ltd) and covered with mineral oil (Nanjing Aibei Biotechnology Co., Ltd) in the petri dish (Ibidi), and culture in a sterile and humid incubator with 5% CO_2_ at 37 °C. For embryos at later stages, embryos were flushed from the oviduct or uterus in M2 medium, and cultured in KSOM medium covering with mineral oil in the petri dish. Embryos were cultured in a sterile and humid incubator with 5% CO_2_ at 37 °C.

### HBimmCue Labeling for Live‐Cell Imaging of Cell Lines, Neurons and Mouse Embryo

The concentration and labeling time largely depend on the sample type, culture conditions and imaging conditions. Briefly, primary cells and thick tissue samples usually require higher staining concentrations and longer staining time duration. For pre‐experiment tests, A 500 nM labeling for 15–30 min was recommended for cells, and 5 µM labeling for 15–30 min for tissues. To obtain images with a high signal‐to‐noise ratio, washing with cell culture medium 2–3 times after labeling was recommended to remove the unbinding probes in the medium. *a) Labeling of cell lines and neurons*: For COS‐7, U‐2OS, BSC1, MELAS and CPEO cell labeling in this study, cells were seeded in glass‐bottom dishes (Cellvis, #1.5H, D35‐20‐1.5H) one day prior to imaging and cultured at 37 °C with 5% CO_2_ and 95% humidity. Cells were stained with full culture medium supplemented with 500 nM HBimmCue at 37 °C for 15 min. For mouse hippocampus neuron labeling, cells were seeded in glass‐bottom dishes at DIV0 and cultured until DIV7 for imaging. Neurons were stained with Neurobasal medium supplemented with 500 nM HBimmCue at 37 °C for 30 min. Following the staining procedure, cells were washed twice with fresh culture for imaging. *b) Labeling of mouse oocytes and pre‐implantation embryos*: For mouse oocytes and pre‐implantation embryos labeling in this study, samples were stained with KSOM medium supplemented with 5 µM HBimmCue at 37 °C for 30 min. Following the staining procedure, embryos were washed with M2 medium for three times and transferred to fresh M2 medium in glass‐bottom dishes for imaging.

### Commercial Dyes

Mito Tracker Green (A66468, Invitrogen), Mito Tracker Deep Red (A66440, Invitrogen), LysoView488 (70067, biotium), ER Tracker Green (E34251, Invitrogen) were used according to the protocol of the products.

### FLIM Imaging

All the single‐photon fluorescence lifetime imaging experiments were performed on the Leica Stellaris 8 system, equipped with a white light laser source operating at 80 MHz pulse repetition rate and a time‐correlated single photon counting (TCSPC) module. A 20× Leica air objective (NA 0.5) was used for embryo imaging, and a 100× Leica oil immersion objective (NA 1.40) was used for living cell imaging. The acquisition parameters of all the experiments are listed in Table S2 (Supporting Information).

### STED Imaging

STED imaging experiments were performed on the Abberior Facility STED microscope or Leica Stellaris 8 microscope. A 100× oil‐immersion objective lens was used for imaging. The acquisition parameters of all the experiments are listed in Table S2 (Supporting Information).

### SIM Imaging

All the SIM imaging experiments were performed on the fully integrated Polar‐SIM super‐resolution microscope imaging system (Airy Technology Co. Ltd)^[^ ^41^ ^]^ A 100× oil‐immersion objective lens was used for imaging. The acquisition parameters of all the experiments are listed in Table S2 (Supporting Information).

### Cellular Viability Assay

COS‐7 cells were plated into the wells of a flat‐bottom 96‐well plate (2 × 10^4^ cells per well in 250 µL of media) and cells were incubated overnight. On the next day, the cells in each well were washed with 1× PBS, followed by incubated in 250 µL cell culture medium supplemented with either 500 nM HBimmCue or 500 nM MitoTrackerGreen for 20 min. After incubation, the media in each well was replaced with 100 µL of DPBS and 100 µL CellTiterGlo (Promega, G7570) solution. The cell plate was incubated at room temperature for 15 min in the dark. Then, the bioluminescent level of each well was measured using a Synergy H1 plate reader (96‐well opaque‐walled tissue culture plate). Wells without cells were used as negative controls, and the relative bioluminescence signals of each well were calculated.

### Cellular mβCD Treatment

COS‐7 cells were seeded in glass‐bottom dishes (Cellvis, #1.5H, D35‐20‐1.5H) one day prior to imaging and cultured at 37 °C with 5% CO_2_ and 95% humidity. Cells were pre‐treated with 1 mM mβCD for 3 h and then stained with full culture medium supplemented with 500 nM HBimmCue at 37 °C for 15 min.

### Imaging Data Processing and Analysis—FLIM Imaging

Fluorescence lifetime decays were fitted by an N‐component multi‐exponential function using LAX software. Mean intensity‐weighted lifetimes were used for calculation and statistical analysis unless otherwise stated. The acquisition and fitting parameters of all the FLIM imaging experiments are listed in Table S3 (Supporting Information).

### Imaging Data Processing and Analysis—SIM and STED Imaging

SIM and STED images were deconvolved using the MRA deconvolution software to further improve the signal‐to‐noise ratio and resolution.^[^ ^21b^ ^]^ The PSF was approximated using the Bessel function based on the pixel size, numerical aperture (NA) and emission wavelength in the imaging process.

### Imaging Data Processing and Analysis—STED‐FLIM Imaging

The fluorescence lifetime maps were acquired upon imaging with the STED system. The intensity images were deconvolved using the MRA deconvolution algorithm, then the deconvolved image was superimposed with the acquired fluorescence lifetime map to generate the final STED‐FLIM pseudo‐color image.

### Statistical Analysis

Origin 2024 was used for statistical analysis. Data were expressed as the mean ± standard error of the mean (SEM), and each experiment was independently conducted at least three times. Two‐tailed unpaired Student's *t*‐test was utilized for statistical comparison. ^***^ *p* < 0.001, ^**^ *p* < 0.01, ^*^ *p* < 0.05, n.s. means no significance.

## Conflict of Interest

B.G. is an inventor of the awarded patent (ZL 202311274339.6) of HBimmCue. Other authors declare no competing interests.

## Author Contributions

P.X., B.G., M.L., and S.G. conceived the project. P.X., B.G., and M.L. supervised the research. B.G. provided HBimmCue probes. S.G. designed and performed the experiments with the assistance of J.S. and X.G.; S.G. analyzed the data, and composed the figures and movies. Y.H. performed SIM, STED and STED‐FLIM image processing. S.G., M.L., and P.X. wrote the manuscript with input from all the authors. M.S. kindly provided mouse embryo samples. Y.Z. and H.Z. kindly provided MELAS and CPEO disease cells.

## Supporting information



Supporting Information

Supplemental Movie 1

Supplemental Movie 2

Supplemental Movie 3

Supplemental Movie 4

## Data Availability

The data that support the findings of this study are available in the supplementary material of this article.
