# Supplementary material for: HBimmCue: A Versatile Fluorescent Probe for Multi‐Scale Imaging of Lipid Polarity and Membrane Order in Inner Mitochondrial Membrane
Source: Adv Sci (Weinh). 2025 Feb 9;12(13):2414343. doi: 10.1002/advs.202414343 (PMC11967834; doi:10.1002/advs.202414343)
Supplement: Supplementary file 1 — Supporting Information [file ADVS-12-2414343-s005.docx]

Supporting Information

**HBimmCue: A Versatile Fluorescent Probe for Multi-scale Imaging of Lipid Polarity and Membrane Order in Inner Mitochondrial Membrane**

*Shu Gao, Jing Sun, Yiwei Hou, Xichuan Ge, Ming Shi, Hongxi Zheng, Yan Zhang, Meiqi Li^*^, Baoxiang Gao^*^ and Peng Xi^*^*

**This PDF file includes:**

Figures S1 to S14

Tables S1 to S3

Legends for movies S1 to S4

Chemical synthesis and characterization of new compounds

**Other Supplementary Materials for this manuscript include the following:**

Movies S1 to S4

**Figure S1**


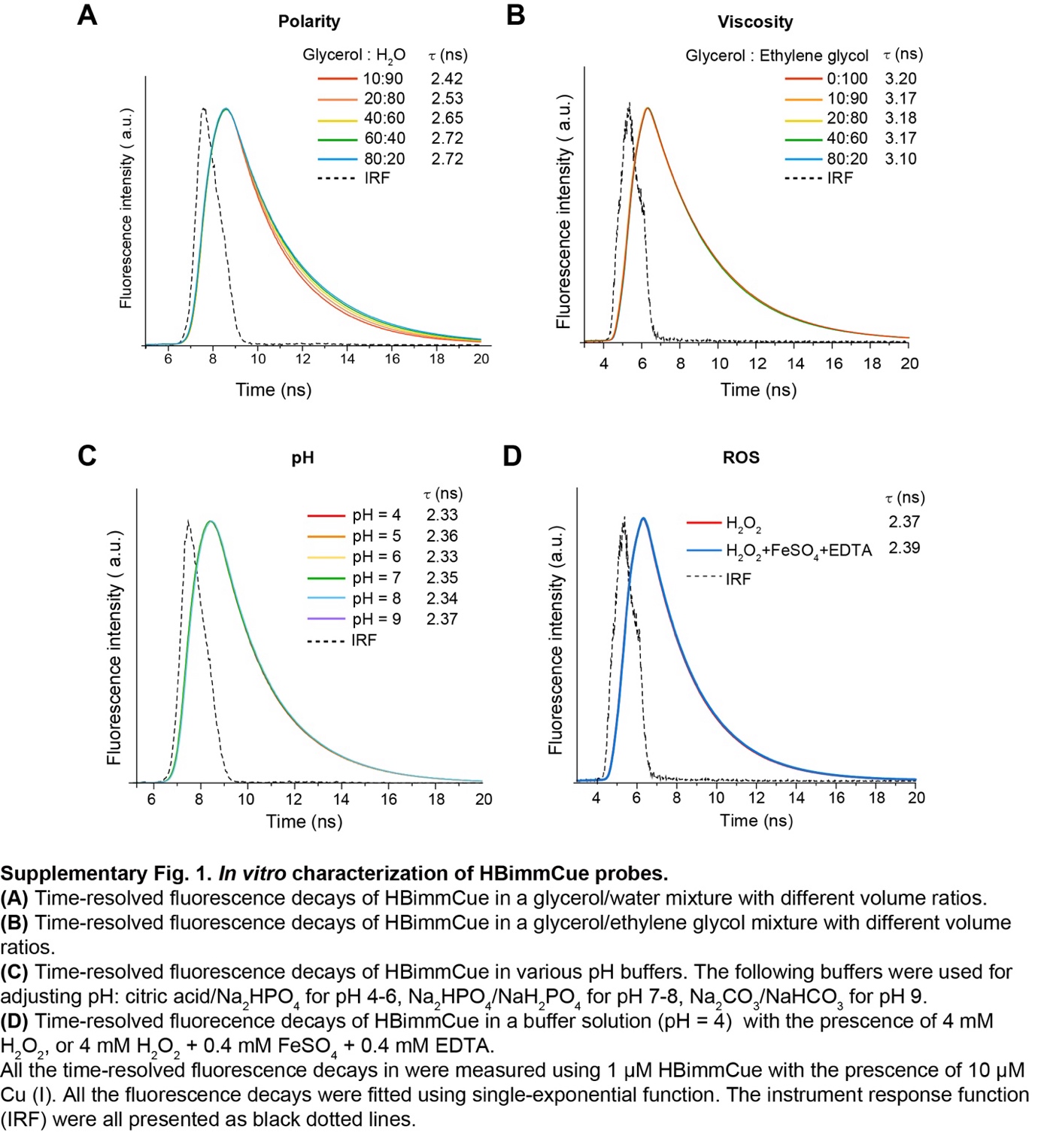


**Figure S1. In vitro characterization of HBimmCue probes.** (A) Time-resolved fluorescence decays of HBimmCue in a glycerol/water mixture with different volume ratios. (B) Time-resolved fluorescence decays of HBimmCue in a glycerol/ethylene glycol mixture with different volume ratios. (C) Time-resolved fluorescence decays of HBimmCue in various pH buffers. The following buffers were used for adjusting pH: citric acid/Na_2_HPO_4_ for pH4-6, Na_2_HPO_4_/NaH_2_PO_4_ for pH7-8, Na_2_CO_3_/NaHCO_3_ for pH9. (D) Time-resolved fluorecence decays of HBimmCue in a buffer solution (pH = 4) with the prescence of 4 mM H_2_O_2_, or 4 mM H_2_O_2_ + 0.4 mM FeSO_4_ + 0.4 mM EDTA. All the time-resolved fluorescence decays in were measured using 1 μM HBimmCue with the presence of 10 μM Cu(I). All the fluorescence decays were fitted using single-exponential function. The instrument response functions (IRF) were all presented as black dotted lines.

**Figure S2**


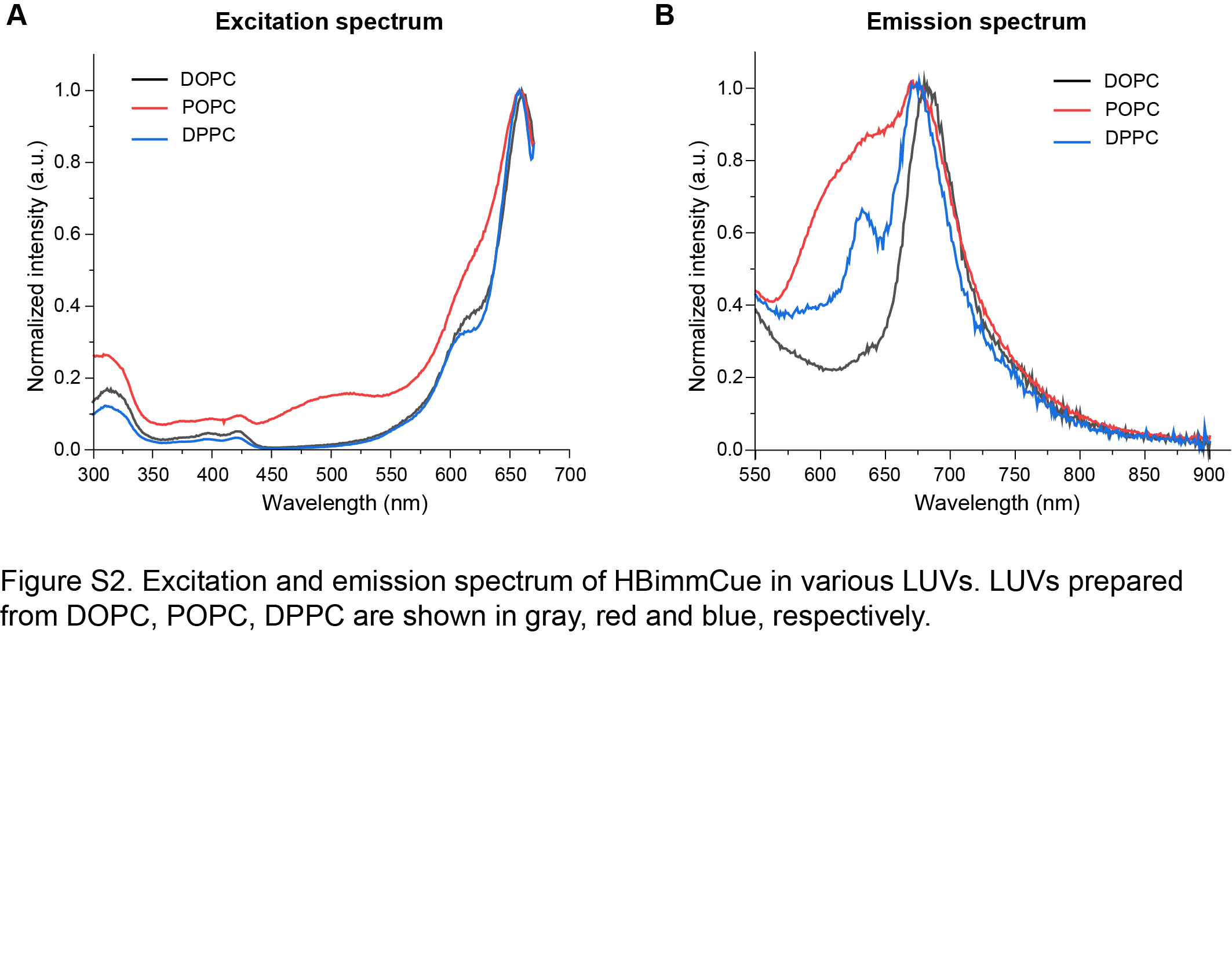


**Figure S2. Excitation and emission spectrum of HBimmCue in various GUVs.** GUVs prepared from DOPC, POPC, DPPC are shown in gray, red and blue, respectively.

**Figure S3**


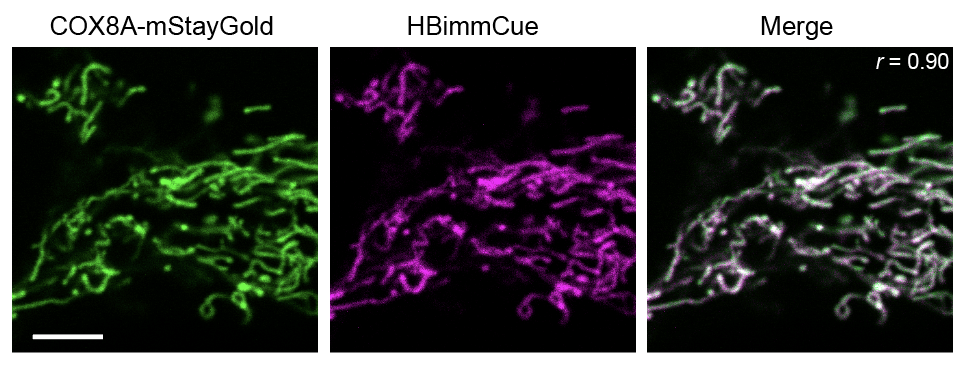


**Figure S3. Co-localization imaging of COS-7 cells labeled with HBimmCue and COX8A-mStayGold.** Representative images of COS-7 cells transfected with COX8A-mStayGold plasmids and then labeled with 500 nM HBimmCue. The Pearson’s correlation factor *(r)* was calculated by the Coloc2 pulgin in Fiji. Scale bars: 5 μm.

**Figure S4**


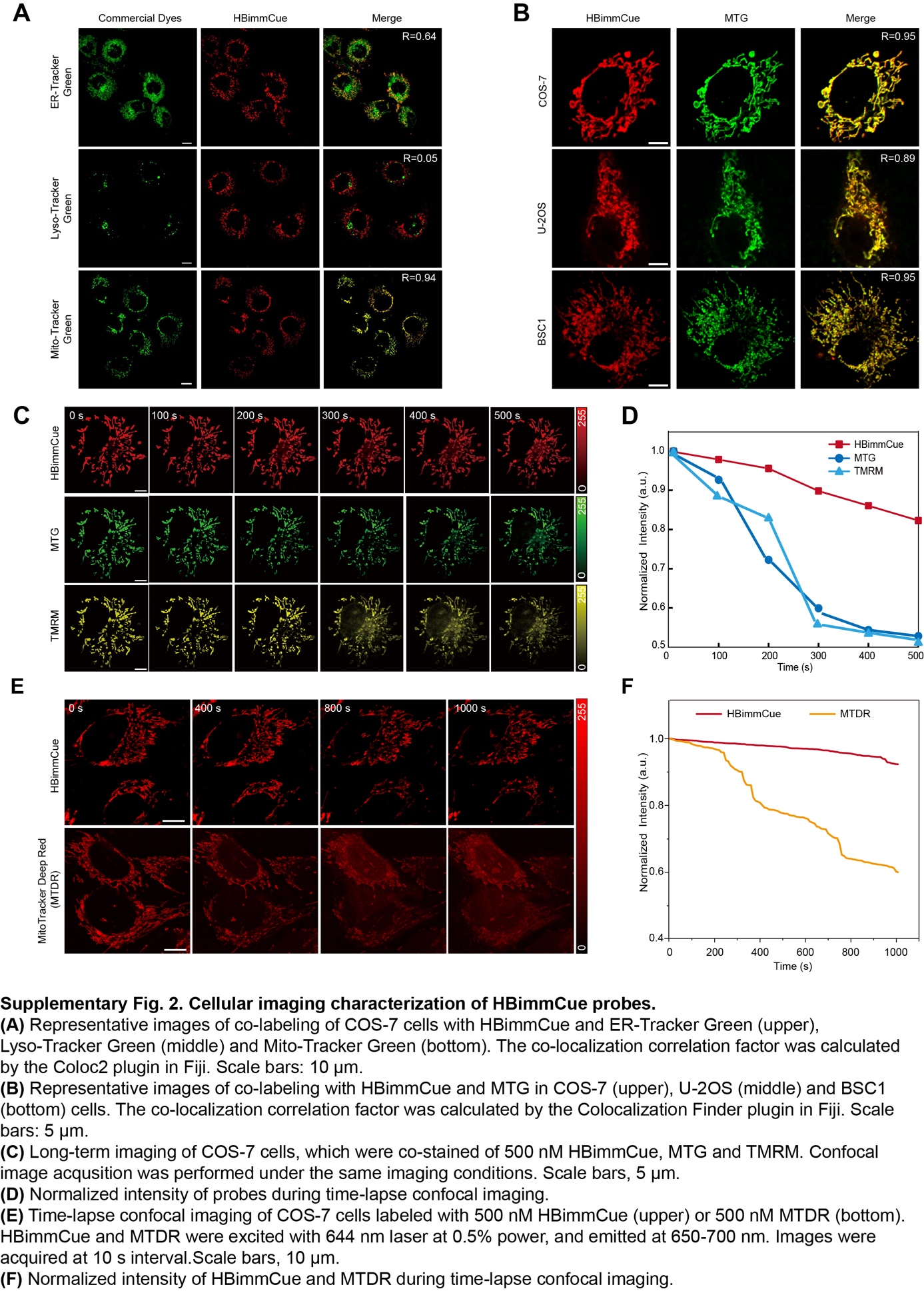


**Figure S4. Cellular imaging characterization of HBimmCue probes.** (A) Representative images of COS-7 cells co-labeled with HBimmCue and ER-Tracker Green (upper), Lyso-Tracker Green (middle) or Mito-Tracker Green (bottom). The Pearson’s correlation factor *(r)* was calculated by the Coloc2 plugin in Fiji. Scale bars: 10 μm. (B) Representative images of co-labeling with HBimmCue and MTG in COS-7 (upper), U-2OS (middle) and BSC1 (bottom) cells. The co-localization correlation factor was calculated by the Colocalization Finder plugin in Fiji. Scale bars: 5 μm. (C) Time-lapse confocal imaging of COS-7 cells co-labeled with 500 nM HBimmCue, MTG and TMRM. Scale bars, 5 μm. (D) Normalized intensity of probes during time-lapse confocal imaging. (E) Time-lapse confocal imaging of COS-7 cells labeled with 500 nM HBimmCue (upper) or 500 nM MTDR (bottom). HBimmCue and MTDR were excited with 644 nm laser at 0.5% power, and emitted at 650-700 nm. Images were acquired at 10 s interval.Scale bars, 10 μm. (F) Normalized intensity of HBimmCue and MTDR during time-lapse confocal imaging.

**Figure S5**

**
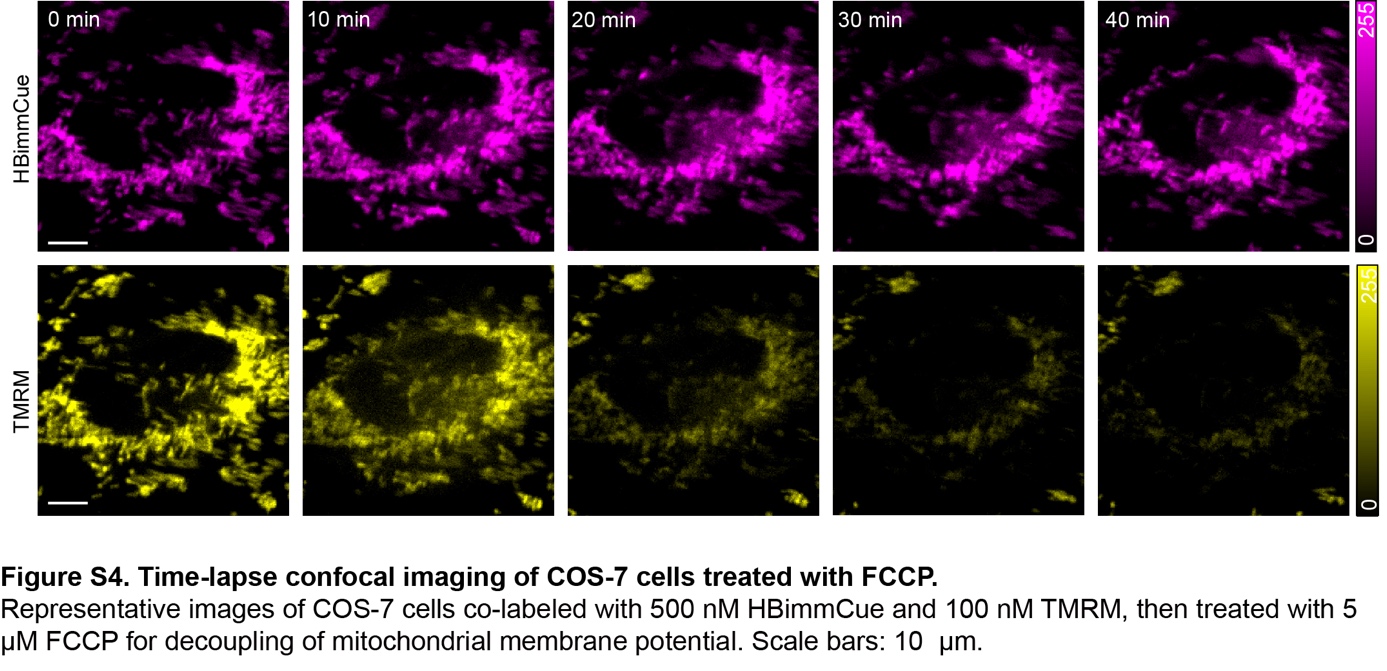
**

**Figure S5. Time-lapse confocal imaging of COS-7 cells treated with FCCP.** Representative images of COS-7 cells co-labeled with 500 nM HBimmCue and 100 nM TMRM, followed by treatment with 5 μM FCCP to disrupt mitochondrial membrane potential. Scale bars: 10 μm.

**Figure S6**

**
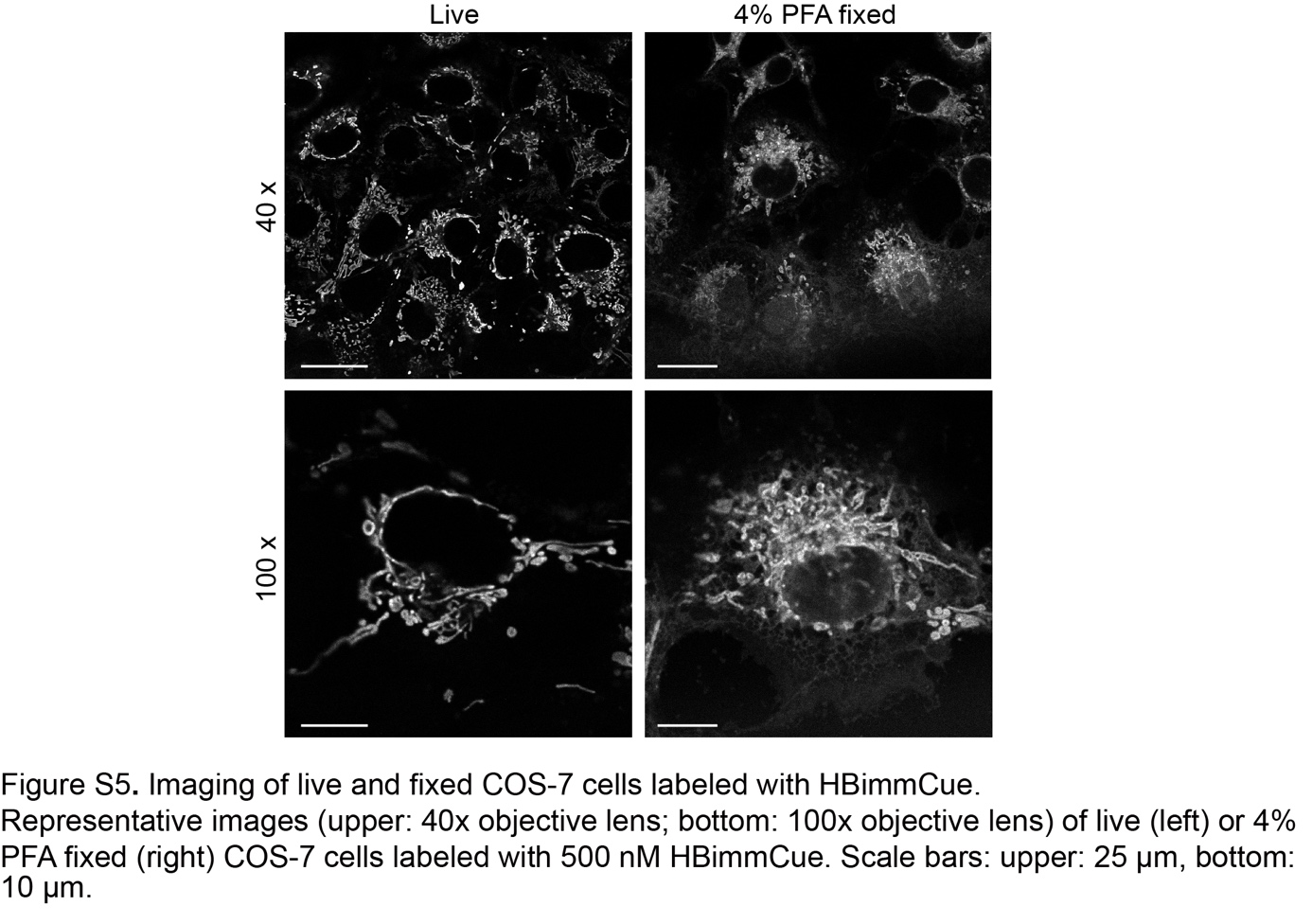
**

**Figure S6. Imaging of live and fixed COS-7 cells labeled with HBimmCue.** Representative images (upper: 40× objective lens; bottom: 100× objective lens) of live (left) or 4% PFA fixed (right) COS-7 cells labeled with 500 nM HBimmCue. Scale bars: upper: 25 μm, bottom: 10 μm.

**Figure S7**


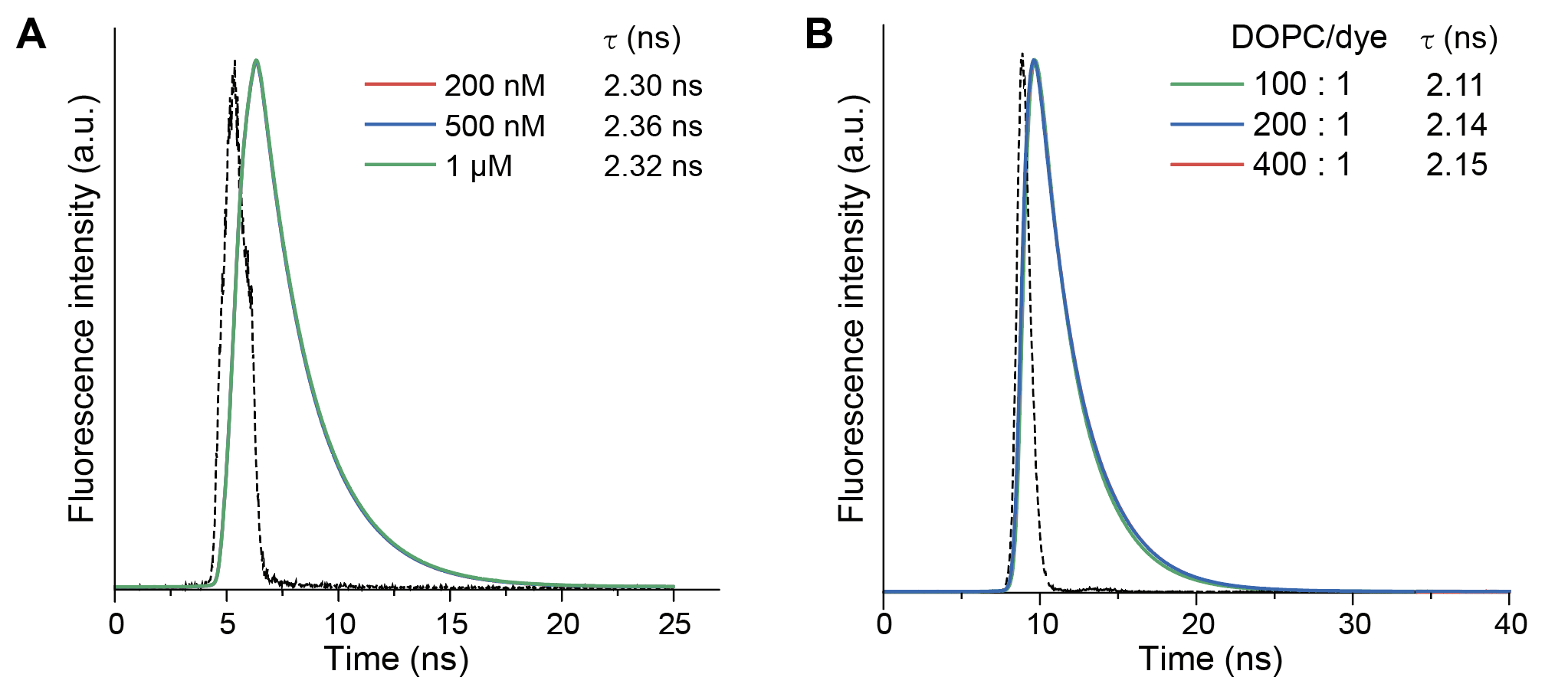


**Figure S7. Fluorescence decays of HBimmCue in solution and GUVs at different concentrations.** (A) Fluorescence decays of HBimmCue in aqueous solution at different concentrations. (B) Fluorescence decays of DOPC/HBimmCue (mol/mol): 100:1 (green), 200:1 (blue), 400:1 (orange). The instrument response function (IRF) is presented as black dotted lines.

**Figure S8**


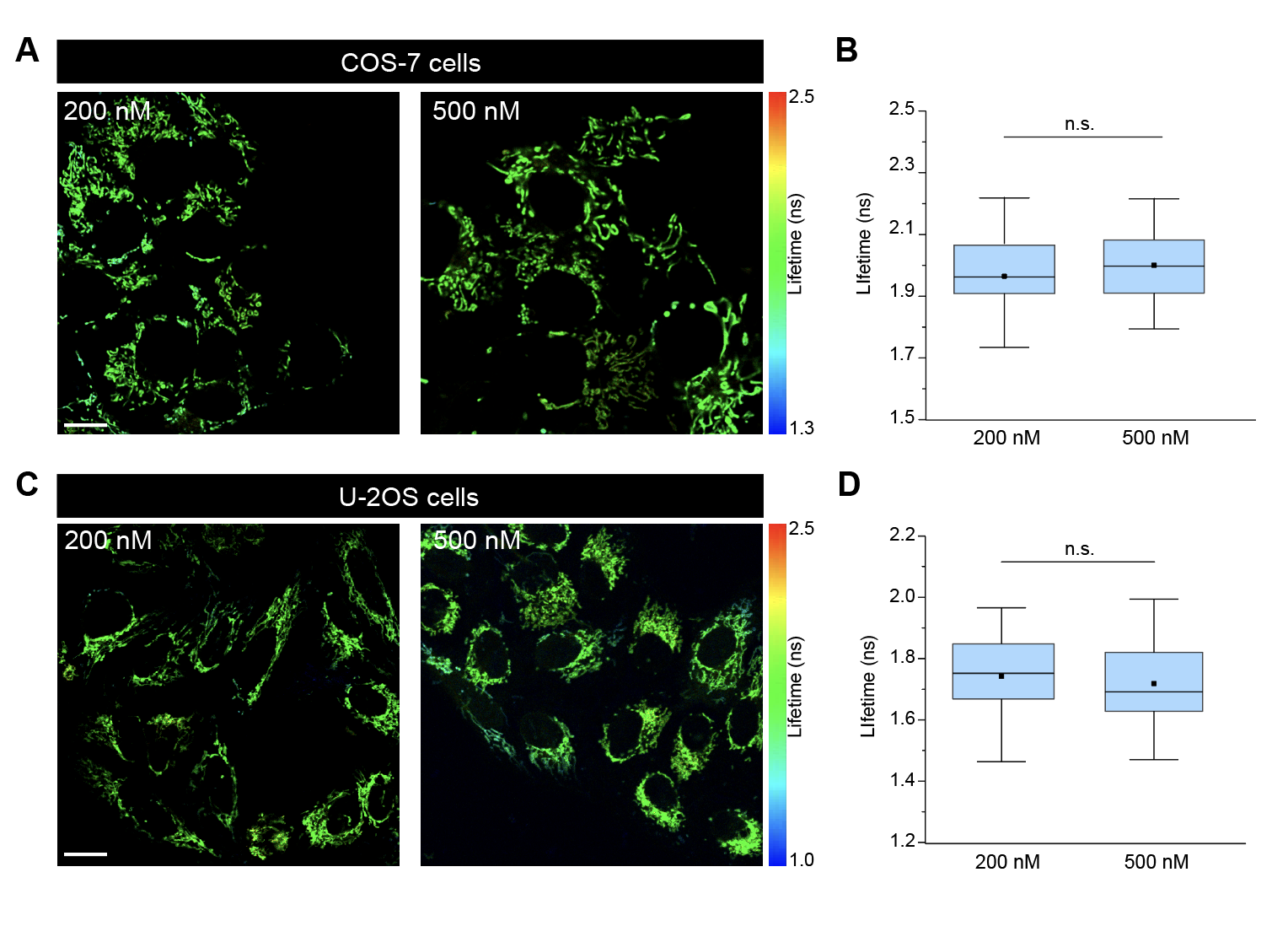


**Figure S8. Fluorescence lifetime imaging of COS-7 cells and U-2OS cells labeled with different concentrations of HBimmCue.** (A)Representative FLIM imaging results of COS-7 cells labeled with 200 nM and 500 nM HBimmCue. Scale bars, 50 μm. (B) Quantitative comparison of mean fluorescence lifetime of HBimmCue in different groups. n = 20 cells per group. (C) Representative FLIM imaging results of U-2OS cells labeled with 200 nM and 500 nM HBimmCue. Scale bars, 50 μm. (D) Quantitative comparison of mean fluorescence lifetime of HBimmCue in different groups. n = 20 cells per group. Mean ± S.D., Two-tailed T-test for the statistic calculation. *P* > 0.05: n.s., *P* < 0.05: *, *P* < 0.01: **; *P* < 0.001: ***.

**Figure S9**

**
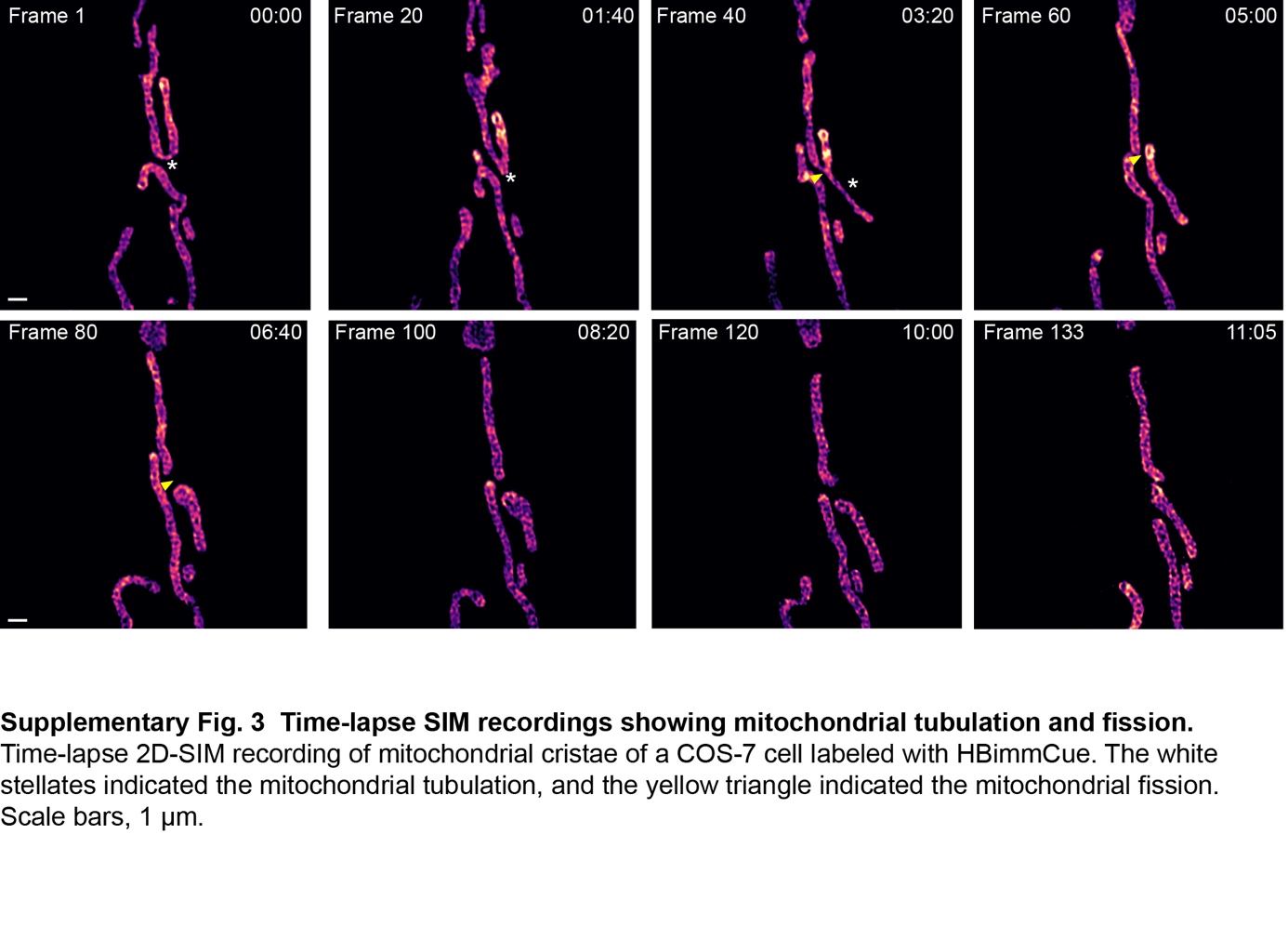
**

**Figure S9. Time-lapse SIM recordings showing mitochondrial tubulation and fission.**

Time-lapse 2D-SIM recording of mitochondrial cristae of a COS-7 cell labeled with HBimmCue. The white stellates indicated the mitochondrial tubulation, and the yellow triangle indicated the mitochondrial fission. Scale bars: 1 μm.

**Figure S10**


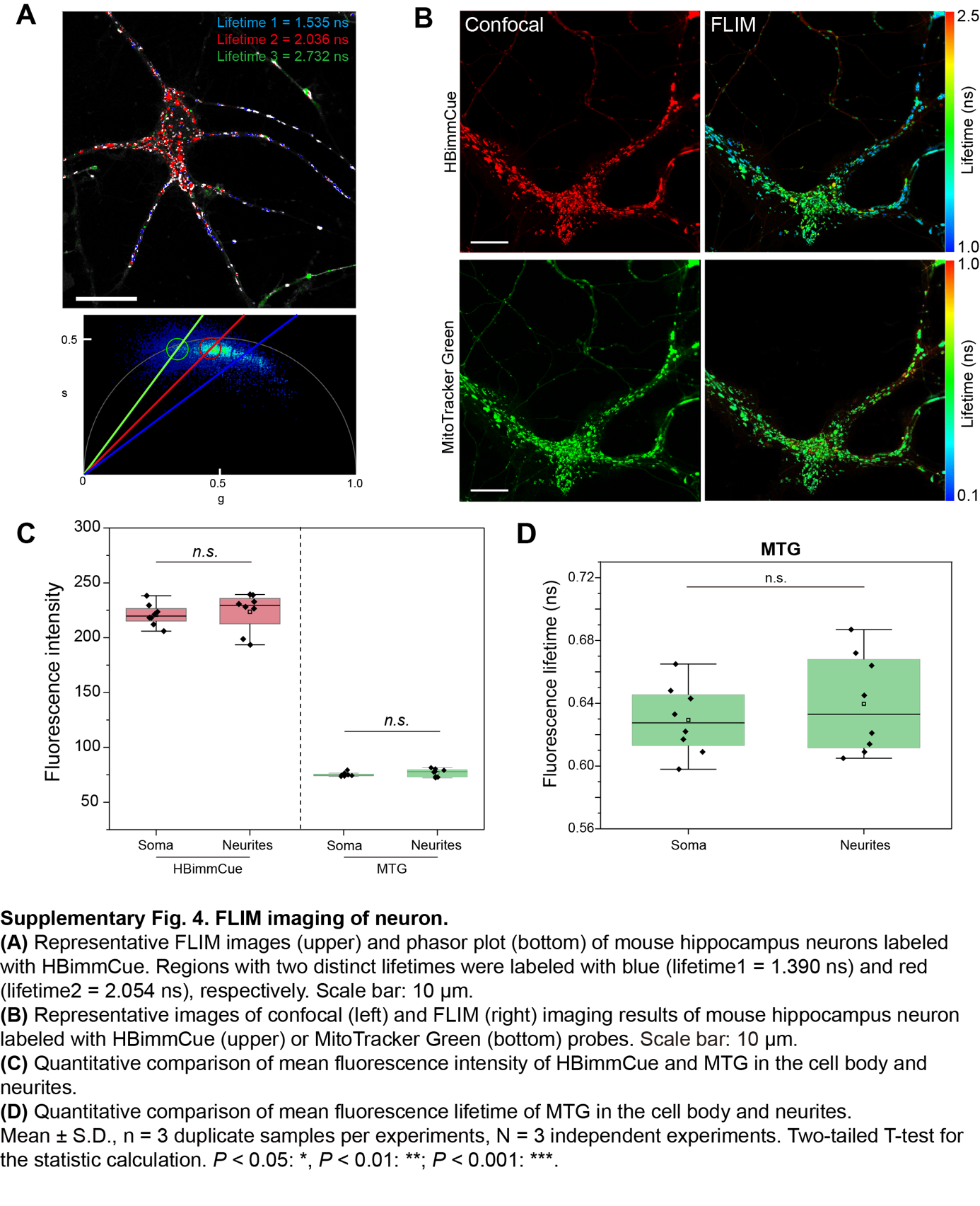


**Figure S10. FLIM imaging of neuron.** (A) Representative FLIM images (upper) and phasor plot (bottom) of mouse hippocampus neurons labeled with HBimmCue. Regions with three distinct lifetimes were labeled with blue (lifetime1 = 1.535 ns), red (lifetime2 = 2.036 ns) and green (lifetime3 = 2.732 ns, background impurities), respectively. Scale bar: 10 μm. (B) Representative images of confocal (left) and FLIM (right) imaging results of mouse hippocampus neuron labeled with HBimmCue (upper) or MitoTracker Green (bottom) probes. Scale bar: 10 μm. (C) Quantitative comparison of mean fluorescence intensity of HBimmCue and MTG in the cell body and neurites. (D) Quantitative comparison of mean fluorescence lifetime of MTG in the cell body and neurites. Mean ± S.D., n = 3 duplicate samples per experiments, N = 3 independent experiments. Two-tailed T-test for the statistic calculation. *P* < 0.05: *, *P* < 0.01: **; *P* < 0.001: ***.

**Figure S11**


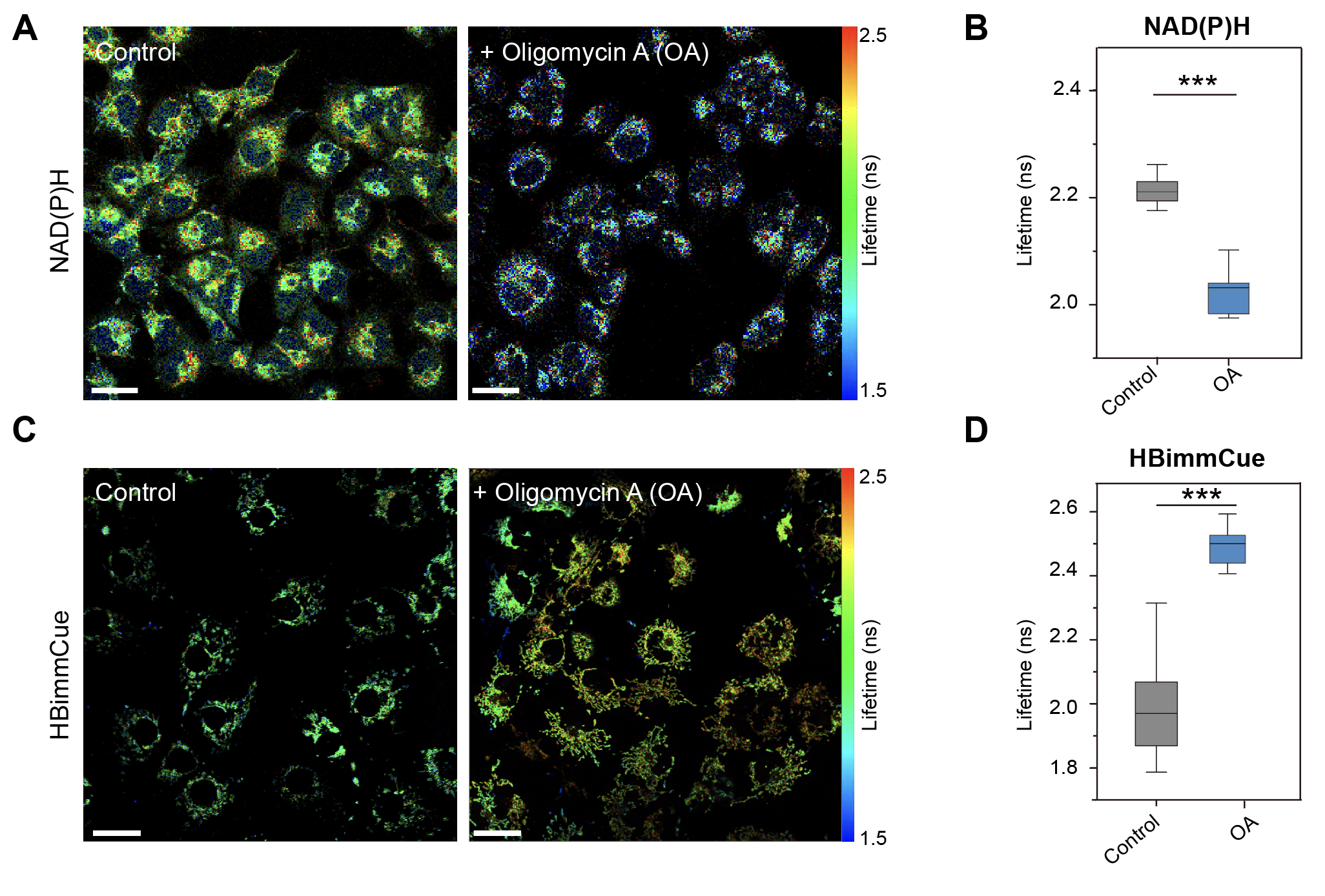


**Figure S11. Fluorescence lifetime of HBimmCue correlates with cellular respiration level.** (A, C) Representative images of NAD(P)H 2p-FLIM imaging results (A) and HBimmCue FLIM imaging results (C) of control (left) and 1.5 μM Oligomycin A-treated (right) COS-7 cells. Scale bars: 50 μm. (B, D) Quantitative comparison of mean fluorescence lifetime of NAD(P)H (B) and HBimmCue (D) in control and Oligomycin A- treated COS-7 cells. Mean ± S.D., n = 3 duplicate samples per experiments, N = 3 independent experiments. Two-tailed T-test for the statistic calculation. *P* < 0.05: *, *P* < 0.01: **; *P* < 0.001: ***.

**Figure S12**


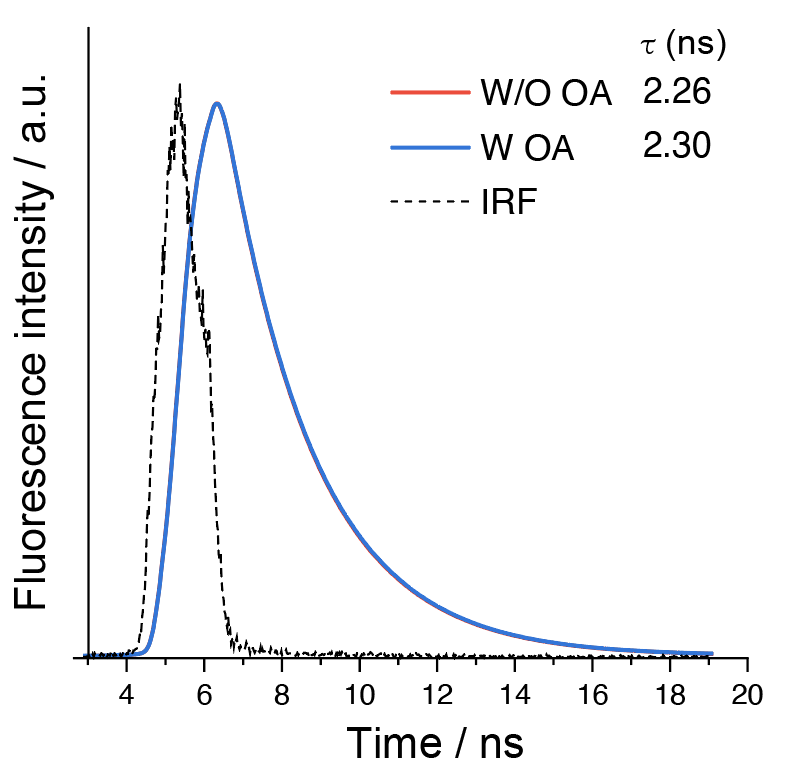


**Figure S12. Influence of Oligomycin A to fluorescence lifetimes of HBimmCue.** Time-resolved fluorescence decays of HBimmCue in aqueous solvent with or without 1 μM Oligomycin A. All the fluorescence decays were fitted using single-exponential function. The instrument response function (IRF) was presented as black dotted lines.

**Figure S13**


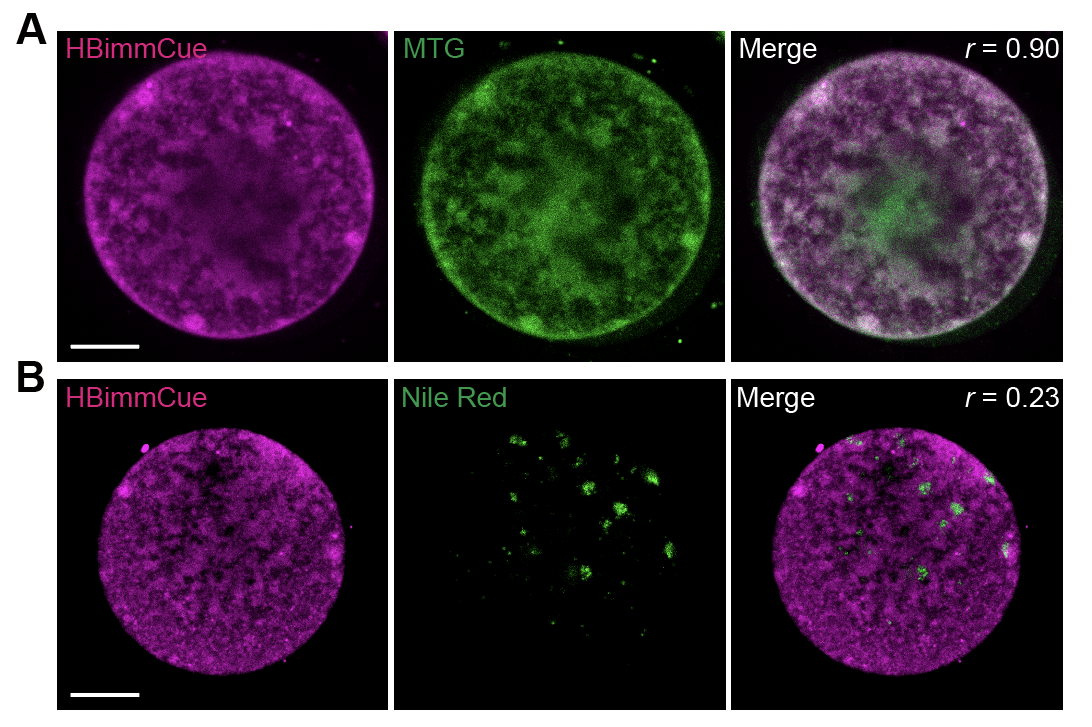


**Figure S13. Confocal imaging of mouse oocytes labeled with HBimmCue and Mito Tracker Green (MTG) or Nile Red.** (A) Representative images of oocytes co-labeled with 5 μM HBimmCue and 5 μM MTG. (B) Representative images of oocytes co-labeled with 5 μM HBimmCue and 5 μg/mL Nile Red. Nile Red was excited with 488 nm laser, and the emission range was 500-550 nm. The Pearson’s correlation factor *(r)* was calculated by the Coloc2 plugin in Fiji. Scale bars: 20 μm.

**Figure S14**


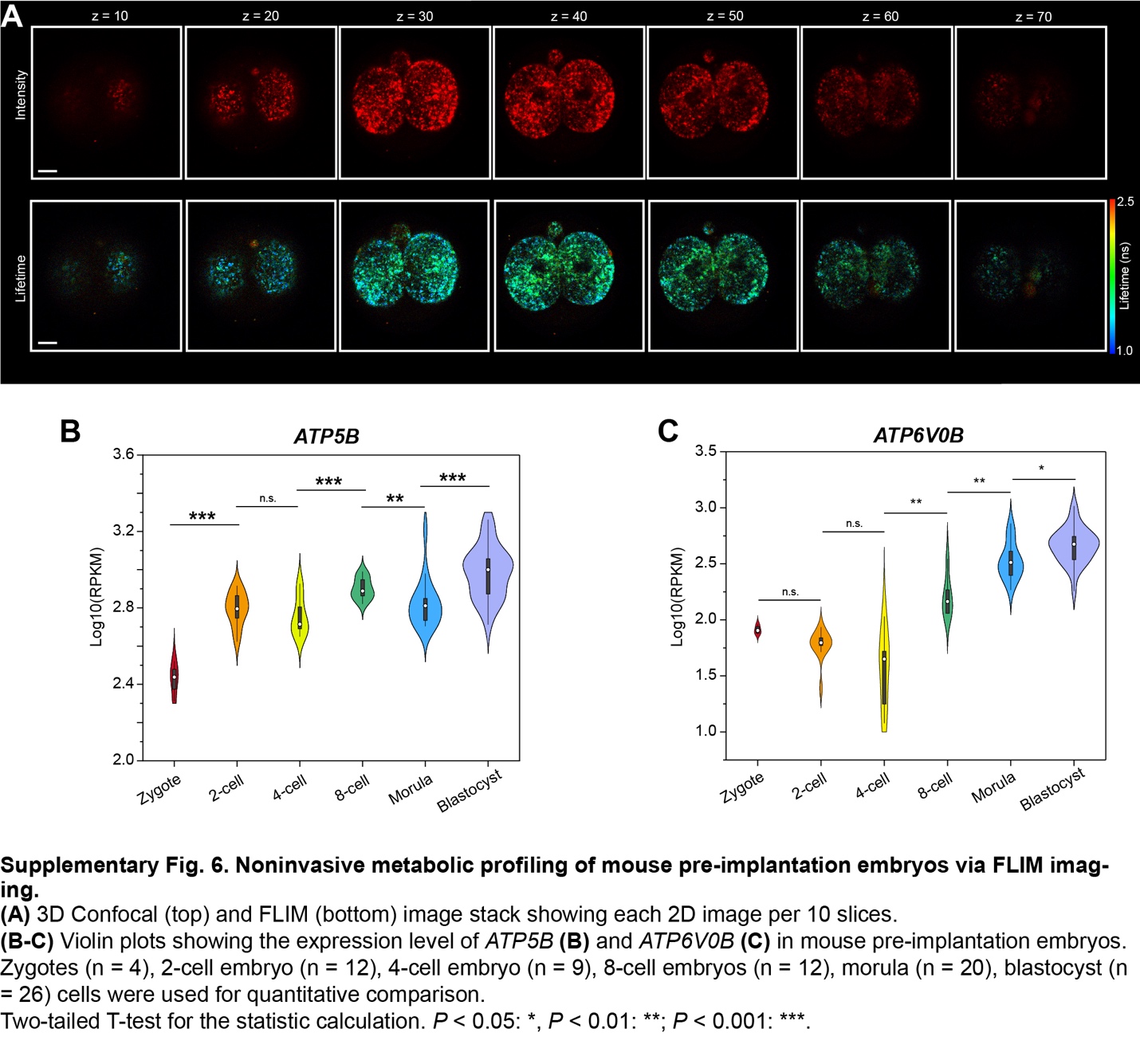


**Figure S14. Noninvasive metabolic profiling of mouse pre-implantation embryos via FLIM imaging.** (A) 3D Confocal (top) and FLIM (bottom) image stack showing each 2D image per 10 slices. Scale bars, 10 μm. (B-C) Violin plots showing the expression level of *ATP5B* **(B)** and *ATP6V0B* **(C)** in mouse pre-implantation embryos. Zygotes (n = 4), 2-cell embryo (n = 12), 4-cell embryo (n = 9), 8-cell embryos (n = 12), morula (n = 20), blastocyst (n = 26) cells were used for quantitative comparison. Two-tailed T-test for the statistic calculation. *P* < 0.05: *, *P* < 0.01: **; *P* < 0.001: ***.

**Table S1 Fluorescence probes target mitochondria.**

| **Name** | **Localization** | **Excitation wavelength (nm)** | **Environment sensitivity** | **Measurement** | **STED imaging** | **STED-FLIM imaging** | **Applications** | **References** |
| --- | --- | --- | --- | --- | --- | --- | --- | --- |
| HBimmCue | IMM | 638 | Polarity | FLIM | Yes | Yes | Cell lines (COS-7, U-2OS, BSC1) | This work |
|  |  |  |  |  |  |  | Primary cells (mouse hippocampus neuron) |  |
|  |  |  |  |  |  |  | Mitochondrial diseases derived cell lines (CPEO, MELAS) |  |
|  |  |  |  |  |  |  | Mouse pre-implantation embryos |  |
| MitoEsq-635 | IMM | 638 | N.A. | Intensity | Yes | N.A. | Cell lines (Hela, MCF7, RAW264.7, U-2OS) | Yang et al., 2020, Nat. Comm.^[1]^ |
|  |  |  |  |  |  |  | Primary cells (Mouse hippocampus neuron) |  |
| **Name** | **Localization** | **Excitation wavelength (nm)** | **Environment sensitivity** | **Measurement** | **STED imaging** | **STED-FLIM imaging** | **Applications** | **References** |
| NRMito | Mitochondria | 561 | Polarity | Spectrum ratio | N.A. | N.A. | Cell lines (KB cells) | Danylchuk et al., 2021, JACS.^[2]^ |
| PKMO | IMM | 561 | N.A. | Intensity | Yes | N.A. | Cell lines (COS-7, Hela, U-2OS) | Liu et al., 2022, PNAS.^[3]^ |
|  |  |  |  |  |  |  | Primary cells (Brown adipocyte, hippocampal neuron) |  |
| Mitorotor-1 | Mitochondria | 561 | Viscosity | FLIM, FRAP | N.A. | N.A. | Cell lines (NIH3T3) | Singh et al., 2023, PNAS.^[4]^ |
| SiRPFA | Mitochondria | 638 | N.A. | Intensity | Yes | N.A. | Cell lines (COS-7, U-2OS) | Sun et al., 2023, Chem. Comm.^[5]^ |
| MAO-SiR | Mitochondria | 638 | N.A. | Intensity | Yes | N.A. | Cell lines (Hela) | Zheng et al., 2024, Nat. Chem. Bio.^[6]^ |
| **Name** | **Localization** | **Excitation wavelength (nm)** | **Environment sensitivity** | **Measurement** | **STED imaging** | **STED-FLIM imaging** | **Applications** | **References** |
| HBmito  Crimson | IMM | 638 | N.A. | Intensity | Yes | N.A. | Cell lines (COS-7) | Ren et al., 2024, Light Sci. Appl.^[7]^ |
| Mito-Laurdan | mitochondria | 405 | Lipid packing | Spectrum ratio | No | No | Cell lines (Hela cells) | Wong et al., 2024, ACS Chem. Bio.^[8]^ |
| MitoPB Red | IMM | 540 | Polarity | FLIM | Yes | Yes | Cell lines (Hela, A431, HuH-7, HepG2) | Wang et al., 2024, Angew. Chem. Int. Ed.^[9]^ |

[a]N.A. not available.

**Table S2** **Fluorescence imaging data acquisition parameters of this study.**

| **Figure** | **Samples** | **Microscope** | **Labels** | **Labeling conditions** | **Imaging mode** | **Excitation laser** | **Depletion**  **laser** | **Objective lens** | **Detection range (nm)** | **Pixel dwell time (μs)** | **Pixel size (nm)** |
| --- | --- | --- | --- | --- | --- | --- | --- | --- | --- | --- | --- |
| 2B | COS-7 | Lecia Stellaris 8 | HBimmCue | 500 nM 15 min DMEM | Confocal | WLL 638 nm (1%) |  | 100 × | 645-740 nm | 2.825 | 80 |
|  |  |  | MitoTrackerGreen | 100 nM 15 min  DMEM |  | WLL 488 nm (5%) |  |  | 495-580 nm |  |  |
| 2E | COS-7 | Abberior Facility line STED | HBimmCue | 500 nM 15 min DMEM | STED | 640 nm (5%) | 775 nm (25%) | 100 × | 745-740 nm | 0.7 | 20 |
| 2G | COS-7 | Lecia Stellaris 8 | HBimmCue | 500 nM 15 min DMEM | STED FLIM | WLL 638 nm (5%) | 775 nm (80%) | 100 × | 645-740 nm | 1.575 | 23 |
| 3A, S3,9,11 | COS-7 | Lecia Stellaris 8 | HBimmCue | 500 nM 15 min DMEM | Confocal FLIM | WLL 638 nm (1%) |  | 100 × | 645-740 nm | 2.825 | 80 |
| 3C-D | COS-7 | Lecia Stellaris 8 | HBimmCue | 500 nM 15 min DMEM | Confocal FLIM | WLL 638 nm (1%) |  | 100 × | 645-740 nm | 2.825 | 80 |
|  |  |  | LysoView488 | 1 μM 15 min DMEM | Confocal | WLL 488 nm (5%) |  |  | 495-580 nm |  |  |
| **Figure** | **Samples** | **Microscope** | **Labels** | **Labeling conditions** | **Imaging mode** | **Excitation laser** | **Depletion**  **laser** | **Objective lens** | **Detection range (nm)** | **Pixel dwell time (μs)** | **Pixel size (nm)** |
| 4A  S10A-B | Neuron | Lecia Stellaris 8 | HBimmCue | 500 nM 30 min Neurobasal | Confocal FLIM | WLL 638 nm (1%) |  | 20 × | 645-740 nm | 1.22 | 120 |
| 4C, D | CPEO  MELAS | Lecia Stellaris 8 | HBimmCue | 500 nM 15 min DMEM | Confocal FLIM | WLL 638 nm (0.5%) |  | 20 × | 645-740 nm | 1.22 | 227 |
| 5, 6, S14 | Oocytes,  Pre-implantation  embryos | Lecia Stellaris 8 | HBimmCue | 5 μM 30 min KSOM | Confocal  FLIM | WLL 638 nm (1%) |  | 20 × | 645-740 nm | 1.22 | 227 |
| S4A | COS-7 | Lecia Stellaris 8 | HBimmCue | 500 nM 15 min DMEM | Confocal | WLL 638 nm (1%) |  | 100 × | 645-740 nm | 2.825 | 80 |
|  |  |  | ER/Lyso/  Mito Tracker Green | 100 nM 15 min DMEM |  | WLL 488 nm (10%) |  |  | 495-580 nm |  |  |
| **Figure** | **Samples** | **Microscope** | **Labels** | **Labeling conditions** | **Imaging mode** | **Excitation laser** | **Depletion**  **laser** | **Objective lens** | **Detection range (nm)** | **Pixel dwell time (μs)** | **Pixel size (nm)** |
| S4B | COS-7  U-2OS  BSC-1 | Lecia Stellaris 8 | HBimmCue | 500 nM 15 min DMEM | Confocal | WLL 638 nm (1%) |  | 100 × | 645-740 nm | 2.825 | 80 |
| S5 | COS-7 | Lecia Stellaris 8 | HBimmCue | 500 nM 15 min DMEM | Confocal | WLL 638 nm (1%) |  | 100 × | 645-740 nm | 2.825 | 80 |
|  |  |  | TMRM | 100 nM 15 min DMEM |  | WLL 540 nm (1%) |  |  | 550-600 nm |  |  |
| S6 | COS-7 | Airy polar SIM | HBimmCue | 500 nM 15 min DMEM | 2D SIM | 647 nm (2%) |  | 100 × | 650-740 nm |  | 32.5 |
| S8A | COS-7 | Leica SP8 Dive | NAD(P)H |  | 2-photon FLM | 720 nm (2%) |  | 10 × | 468 nm |  | 300 |

**Table S3 Fluorescence lifetime fitting parameters for cellular imaging results.**

| **Figure** | | **Number of photons** | **Fitting model** | **Tau1 (ns)** | **Tau2 (ns)** | **Tau3 (ns)** | **Intensity1 (kCnts)** | **Intensity2**  **(kCnts)** | **Intensity3**  **(kCnts)** | **Intensity weighted mean tau (ns)** | **Chisquares** |
| --- | --- | --- | --- | --- | --- | --- | --- | --- | --- | --- | --- |
| 2g | | 14,927,853 | tri-exponential | 0.102 | 1.097  ±0.064 | 2.462  ±0.045 | 3,539.476  ±14.558 | 1,918.597  ±243.296 | 3,815.656  ±18.063 | 1.594  ±0.009 | 1.573 |
| 3a | control | 13,572,770 | bi-exponential | 0.525±0.042 | 2.311±0.026 |  | 1,587.970±107.353 | 11,278.092±103.314 |  | 2.091±0.014 | 1.062 |
|  | mbcd | 14,162,979 | bi-exponential | 0.530±0.062 | 1.979±0.030 |  | 1,898.846±203.763 | 11,566.263±213.256 |  | 1.775±0.014 | 0.952 |
| 3d | | 6,881,293 | tri-exponential | 0.322±0.124 | 1.028±0.198 | 2.431±0.299 | 404.192±320.397 | 2,898.654±434.133 | 3,205.220±722.762 | 1.675±0.036 | 1.87 |
| 4a | | 1,245,251 | tri-exponential | 0.455±0.111 | 1.920±0.784 | 3.562±0.867 | 94.894±16.359 | 697.107±451.729 | 406.282±482.838 | 2.361±0.025 | 1.11 |
| 4c | ctrl | 3,241,557 | bi-exponential | 0.596±0.082 | 2.603±0.030 |  | 196.421±25.600 | 2,891.809±21.109 |  | 2.476±0.018 | 1.365 |
|  | mutant | 2,191,349 | bi-exponential | 0.578±0.060 | 2.739±0.027 |  | 111.685±13.284 | 1,976.653±9.233 |  | 2.624±0.017 | 0.845 |
| 4e, ctrl | | 3,915,837 | bi-exponential | 0.641±0.056 | 2.689±0.026 |  | 236.326±25.389 | 3,504.346±20.515 |  | 2.560±0.015 | 1.236 |
| **Figure** | | **Number of photons** | **Fitting model** | **Tau1 (ns)** | **Tau2 (ns)** | **Tau3 (ns)** | **Intensity1 (kCnts)** | **Intensity2**  **(kCnts)** | **Intensity3**  **(kCnts)** | **Intensity weighted mean tau (ns)** | **Chisquares** |
| 4e, mutant | | 7,384,229 | bi-exponential | 0.749±0.125 | 2.827±0.033 |  | 408.134±58.934 | 6,649.868±57.920 |  | 2.707±0.022 | 0.968 |
| 5a | Young | 8,324,253 | tri-exponential | 0.339±0.012 | 1.132±0.023 | 1.943±0.121 | 546.674±76.435 | 2343.456±123.543 | 5435.563±463.414 | 1.558±0.020 | 0.924 |
|  | Aged | 8,962,380 | tri-exponential | 0.379±0.019 | 1.262±0.127 | 2.635±0.121 | 723.541±75.214 | 2483.204±484.282 | 5372.548±527.898 | 2.047±0.020 | 0.918 |
| 6b | Zygotes | 5,038,404 | tri-exponential | 0.659±0.117 | 1.098±0.034 | 2.312±0.543 | 691.421±41.988 | 1779.058±50.498 | 2564.656±108.834 | 2.133±0.026 | 1.099 |
|  | 2cell | 7,863,588 | tri-exponential | 0.294±0.011 | 1.004±0.049 | 2.551±0.067 | 784.780±56.531 | 2684.400±137.631 | 3951.164±170.287 | 1.753±0.014 | 0.864 |
|  | 4cell | 1,554,353 | tri-exponential | 0.070±0.056 | 0.531±0.195 | 2.607±0.068 | 35.355±24.575 | 129.392±15.418 | 1288.750±25.627 | 2.361±0.029 | 1.27 |
|  | 8cell | 2,803,789 | tri-exponential | 0.318±0.033 | 1.254±0.291 | 2.581±0.141 | 134.582±25.724 | 450.275±244.133 | 2102.609±255.312 | 2.245±0.026 | 0.866 |
|  | Morula | 2,580,199 | tri-exponential | 0.376±0.016 | 1.936±0.017 | 3.591±0.036 | 215.690±9.095 | 1694.355±8.888 | 568.955±8.372 | 2.180±0.008 | 1.326 |
|  | blastocyst | 3,157,794 | tri-exponential | 0.340±0.029 | 1.188±0.235 | 2.474±0.165 | 193.512±34.469 | 648.055±297.589 | 2178.320±315.092 | 2.062±0.026 | 0.968 |

**Legends for Movies S1 to S4**

**
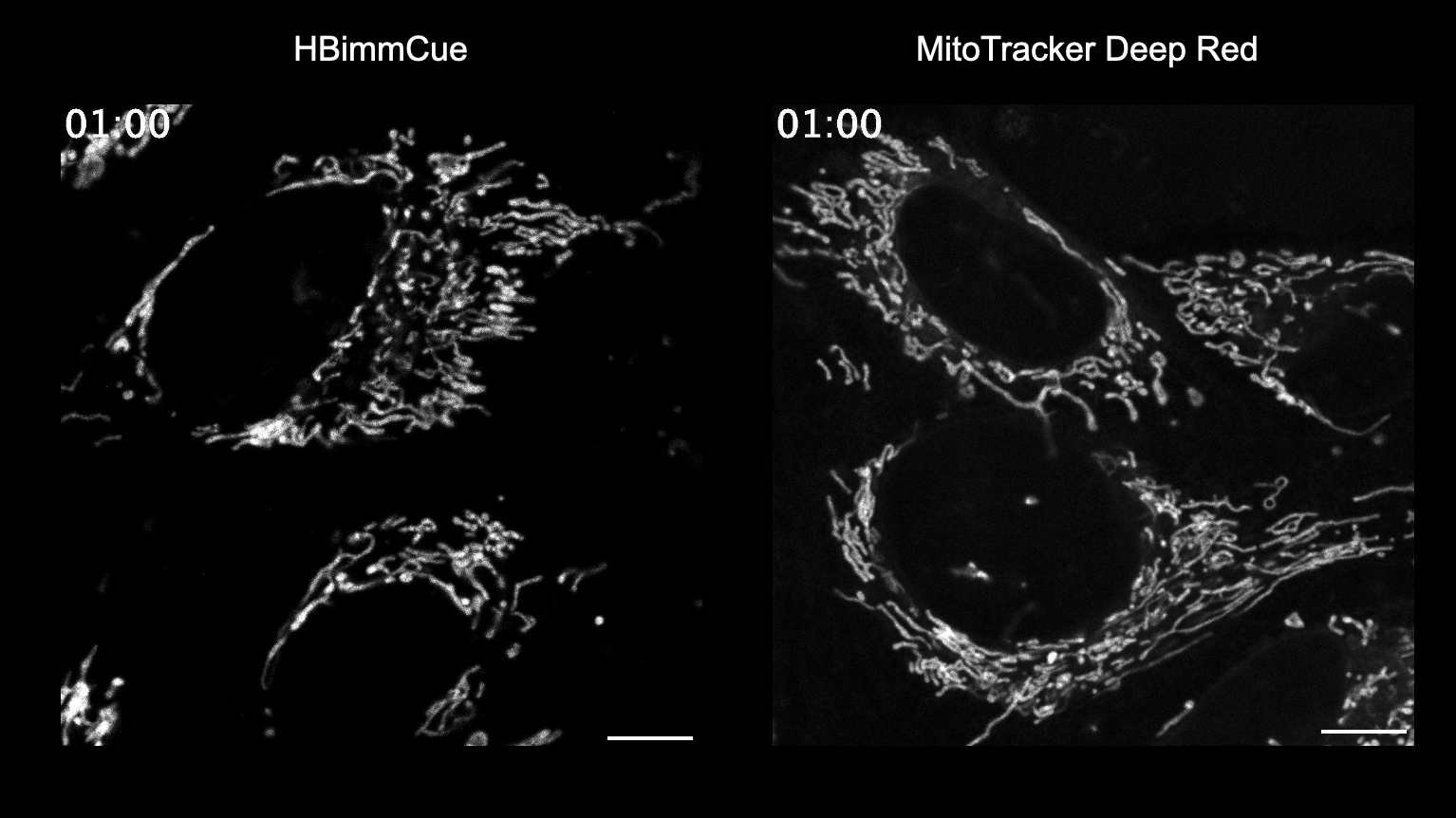
**

**Movie S1.** Time-lapse imaging of COS-7 cells labeled with HBimmCue and MitoTracker Deep Red.

**
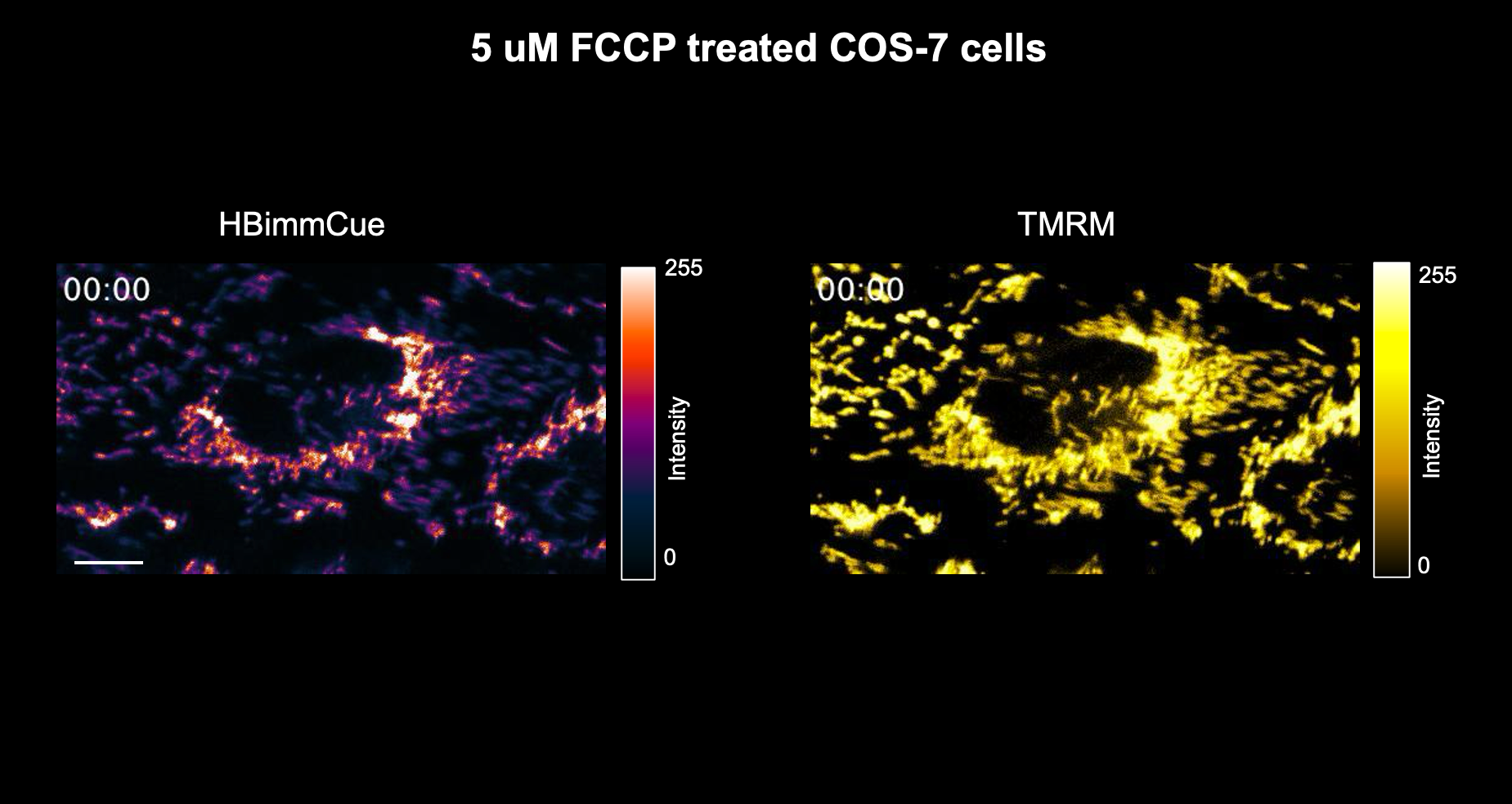
**

**Movie S2.** Time-lapse imaging of COS-7 cells treated with FCCP.


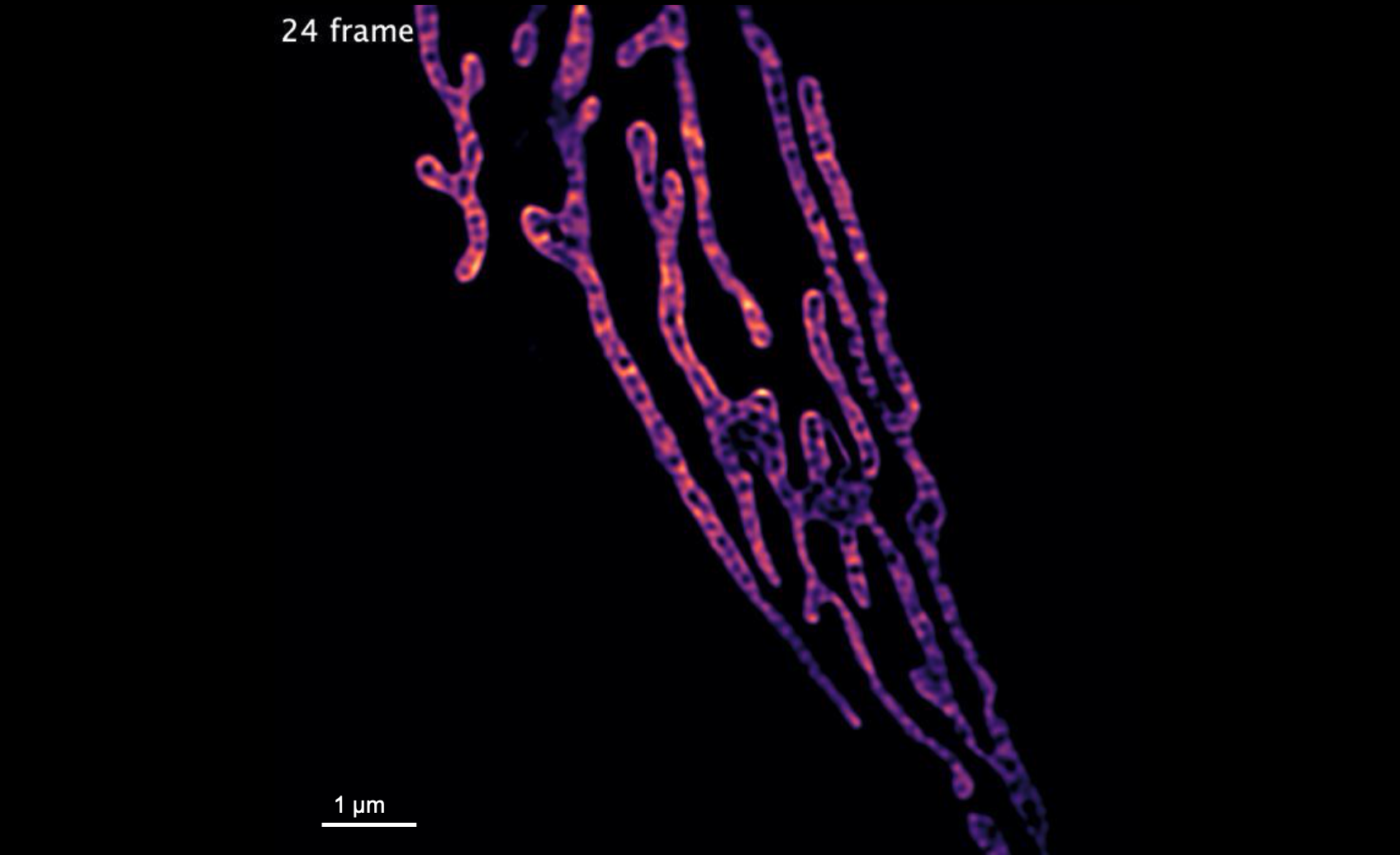


**Movie S3.** Time-lapse SIM imaging of mitochondrial inner membrane in COS-7 cells.

**
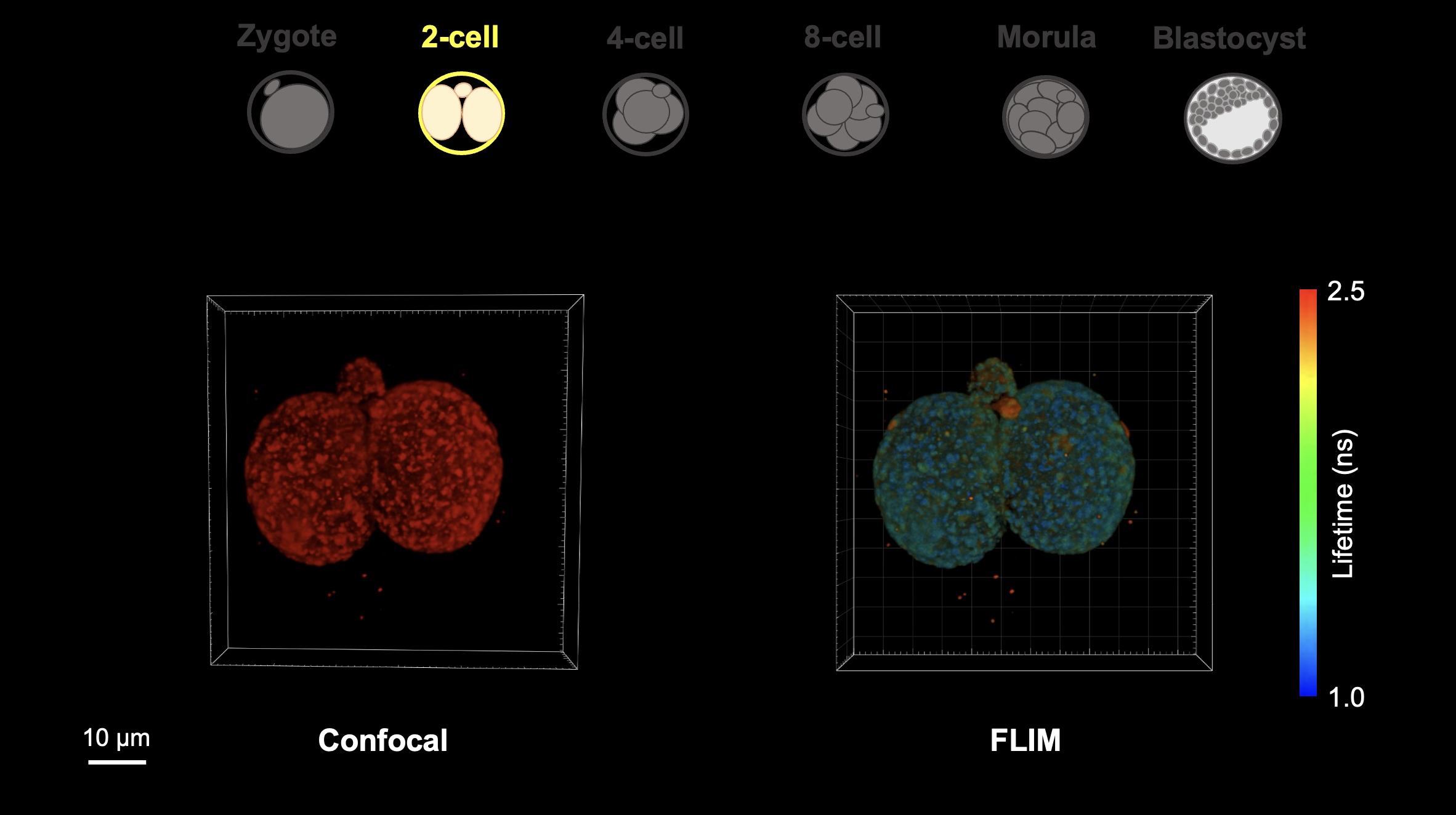
**

**Movie S4.** Fluorescence lifetime imaging of mouse pre-implantation embryos labeled with HBimmCue.

**Chemical synthesis and characterization of HBimmCue**

**Synthesis of HBimmCue**

Synthesis processes of HBimmCue were demonstrated in Scheme S1. All chemical reagents and solvents for synthesis were purchased from commercial suppliers (J&K Chemicals, Energy Chemical and Sigma-Aldrich Chemical) and were used without further purification. All solvents for HPLC were purchased from MREDA. All moisture-sensitive reactions were carried out under an atmosphere of nitrogen. The composition of mixed solvents given as volume ratio (v/v). ^1^H NMR and ^13^C NMR spectra were recorded on an AVANCE III 400 Nanobay (Bruker, 400 MHz for ^1^H, 100 MHz for ^13^C) at room temperature. All chemical shifts (δ) reported in ppm are relative to internal standard tetramethylsilane (δ = 0.0 ppm), or relative to the signals of residual solvent CDCl_3_ (7.26 ppm for ^1^H, 77.16 ppm for ^13^C), and coupling constants are given in Hz. Mass spectra (MS) were measured on a MicroTOF (Bruker) with ESI-TOF (electron spray ionization–time-of-flight). HPLC purification were performed on a Shimadzu PC-31 system equipped with a reverse-phase column (WHAT’POSSIBLE Science, Sunfire Prep C18 10 mm × 150 mm). Eluent A (H_2_O with 1% CH_3_CN) and eluent B (CH_3_OH).


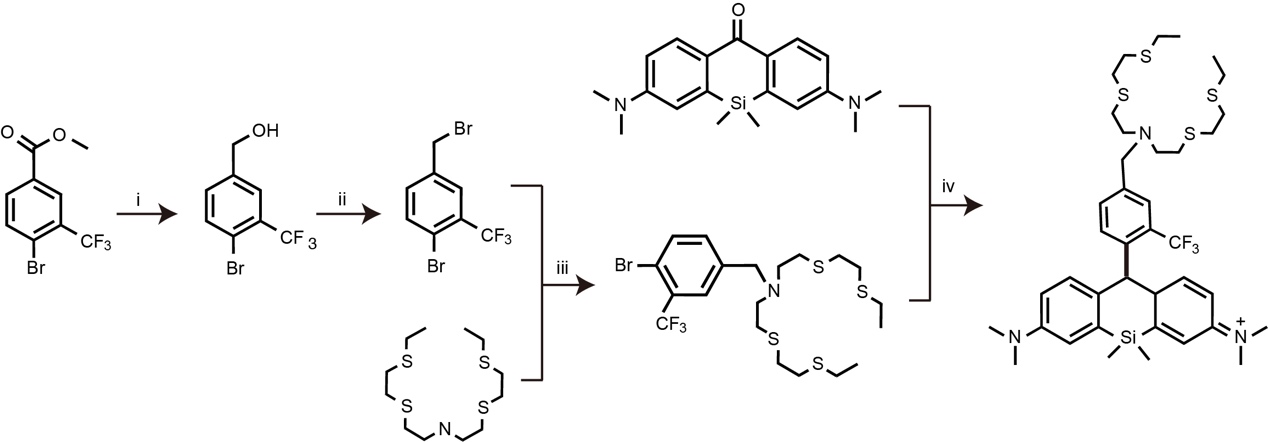


**Scheme S1.** Synthesis of **HBimmCue**. i. LiAlH_4_, THF, 0℃ to r.t. ii. Br_2_OS, CH_3_CN, 45℃. iii. K_2_CO_3_, KI, CH_3_CN, 90℃. iv. tert-BuLi, -78℃ to r.t.

**HBimmCue**


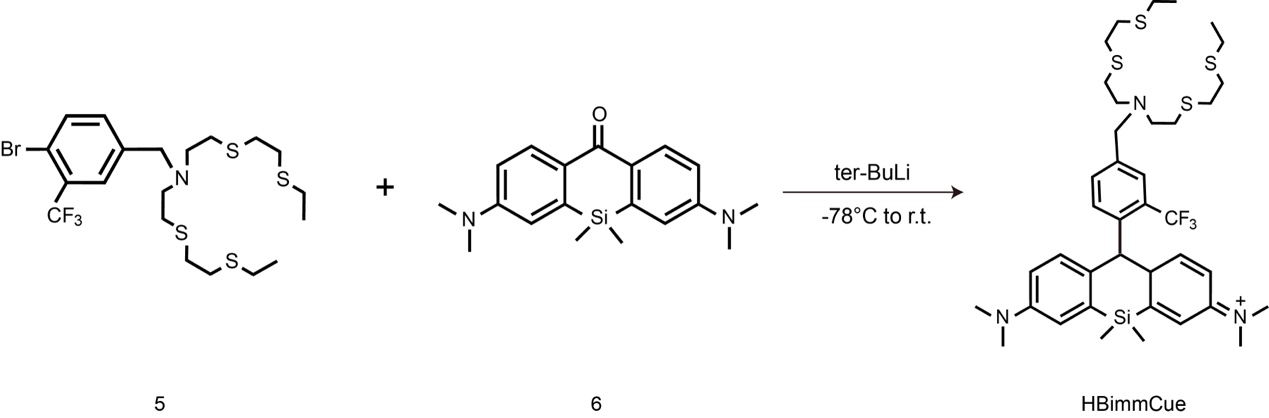


An oven-dried, two-neck round-bottom flask was charged with **5** (0.42 g, 0.76 mmol), and anhydrous THF (7 mL). The solution was cooled to −78°C for 20 min and treated with a solution of tert-BuLi in pentane (1.3 M, 0.59 mL, 0.76 mmol) under a N_2_ atmosphere. After stirring at the same temperature for 2 h, a solution of compound **6** (24.76 mg, 0.076 mmol) in anhydrous THF (3 mL) was added dropwise. The reaction was warmed to r.t., stirred for a 1 h, and then quenched with HCl (2 M, 2 mL). The reaction was poured into saturated (sat.) NaHCO_3_ (20 mL) and extracted with EtOAc (3 ×30 mL). The combined organic layers were washed with brine (30 mL), dried over Na_2_SO_4_, filtered, evaporated and concentrated. The crude residue was purified by silica gel chromatography (CH_2_Cl_2_/CH_3_OH,50:1 to 10/1, v/v), and the residue was further purified by semi-preparative HPLC using eluent A (H_2_O with 1% CH_3_CN) and eluent B (CH_3_OH) (A/B = 80/20 to 0/100 for 40 min) to give a blue solid.

**HBimmCue:** ^1^H NMR (400 MHz, Chloroform-*d*) δ 7.91 (s, 1H), 7.75 (s, 1H), 7.22 (s, 1H), 7.19 (s, 2H), 6.91 (d, *J* = 9.6 Hz, 2H), 6.67 (d, *J* = 12.2 Hz, 2H), 3.85 (s, 2H), 3.40 (s, 12H), 2.85 (s, 4H), 2.73 (s, 12H), 2.56 (d, *J* = 14.8 Hz, 4H), 1.24 (s, 6H), 0.67 (s, 3H), 0.53 (s, 3H).; ^13^C NMR (100 MHz, Chloroform-*d*) δ 165.59, 153.95, 148.17, 141.58, 130.95, 127.95, 113.92, 53.82, 41.09, 32.52, 31.75, 26.03, 14.74.; MS: calc. for C_39_H_55_F_3_N_3_S_4_Si^+^ [M]^+^ 778.2995; found: 778.4900.

^1^H NMR spectrum of **HBimmCue** in CDCl_3_.

^13^C NMR spectrum of **HBimmCue** in CDCl_3_.


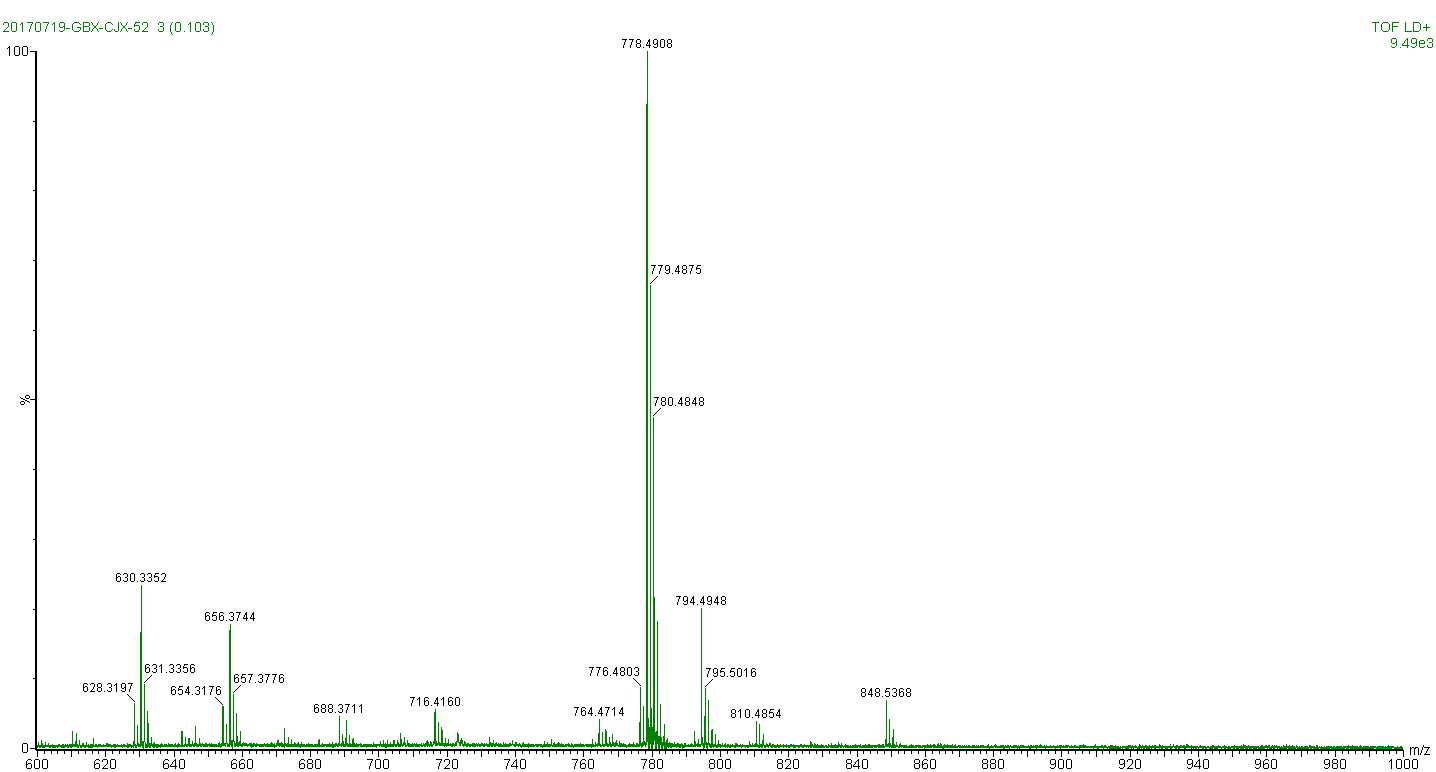


MS spectrum of **HBimmCue**

**References**

[1] X. Yang, Z. Yang, Z. Wu, Y. He, C. Shan, P. Chai, C. Ma, M. Tian, J. Teng, D. Jin, W. Yan, P. Das, J. Qu, P. Xi, *Nat Commun* **2020**, 11, 3699.

[2] D. I. Danylchuk, P. H. Jouard, A. S. Klymchenko, *J Am Chem Soc* **2021**, 143, 912.

[3] T. Liu, T. Stephan, P. Chen, J. Keller-Findeisen, J. Chen, D. Riedel, Z. Yang, S. Jakobs, Z. Chen, *Proc Natl Acad Sci U S A* **2022**, 119, e2215799119.

[4] G. Singh, G. George, S. O. Raja, P. Kandaswamy, M. Kumar, S. Thutupalli, S. Laxman, A. Gulyani, *Proc Natl Acad Sci U S A* **2023**, 120, e2213241120.

[5] J. Sun, X. Ge, B. Jin, S. Li, Y. Hou, S. Zhong, Z. Yang, P. Xi, M. Li, B. Gao, *Chemical Communications* **2023**, 59, 13038.

[6] S. Zheng, N. Dadina, D. Mozumdar, L. Lesiak, K. N. Martinez, E. W. Miller, A. Schepartz, *Nat Chem Biol* **2024**, 20, 83.

[7] W. Ren, X. Ge, M. Li, J. Sun, S. Li, S. Gao, C. Shan, B. Gao, P. Xi, *Light Sci Appl* **2024**, 13, 116.

[8] A. M. Wong, I. Budin, *ACS Chem Biol* **2024**, 19, 1773.

[9] J. Wang, M. Taki, Y. Ohba, M. Arita, S. Yamaguchi, *Angew Chem Int Ed Engl* **2024**, 63, e202404328.
